# Elucidation of Proteoforms of Chinese Hamster Ovary (CHO) Phospholipase B‐Like 2 (PLBL2) Captured From a Monoclonal Antibody

**DOI:** 10.1002/bit.70104

**Published:** 2025-11-10

**Authors:** Michael E. Dolan, Lei (Leo) Wang, Alexander Tedeschi, Yan Wang, Christopher Barton, Sheldon F. Oppenheim, Zhaohui Sunny Zhou

**Affiliations:** ^1^ Department of Chemistry and Chemical Biology Northeastern University Boston Massachusetts USA; ^2^ Barnett Institute for Chemical and Biological Analysis Northeastern University Boston Massachusetts USA; ^3^ Biotherapeutics Process Development Takeda Development Center Americas Cambridge Massachusetts USA; ^4^ Analytical Development, Takeda Development Center Americas Cambridge Massachusetts USA

**Keywords:** charge variants, glycosylation, host cell protein (HCP), phospholipase B‐like 2 (PLBL2), phosphorylation, proteoform, size variants

## Abstract

Removing host cell proteins (HCPs) during biotherapeutic manufacturing is essential to ensure patient safety and supply continuity. Yet, this goal remains exceptionally challenging for some HCP species. For example, phospholipase B‐like‐2 (PLBL2) from Chinese hamster ovary (CHO) cells plagues process engineers by evading typical purification strategies. Separation challenges for CHO PLBL2 and other HCPs are due in large part to a dearth of detailed biochemical characterization for HCPs, such as their proteoforms or variants. Herein, we present as a case study the elucidation of proteoforms of PLBL2, which was endogenously expressed in CHO alongside pembrolizumab (a monoclonal IgG4 antibody) and inadvertently co‐purified following a typical downstream process. Using site‐specifically modified polyclonal anti‐CHO PLBL2 antibodies immobilized onto a solid support, CHO PLBL2 was captured from pembrolizumab, enriched, and recovered, enabling deep characterization by mass spectrometry and other techniques. These analyses revealed a detailed understanding of CHO PLBL2's proteoforms, including charge variants, N‐linked and O‐linked glycosylation, phosphorylation, and cleavage to afford multiple molecular weight isoforms. To the best of our knowledge, this is the first published, deep characterization of a challenging CHO HCP. Armed with a greater understanding of the impurities that plague purification processes, we can design new, targeted strategies to ensure their removal and safeguard both patient safety and biotherapeutic supplies. While our work centered on CHO PLBL2, our antibody immobilization, affinity enrichment, and characterization approach should be broadly applicable to enable the examination of innumerable challenging HCPs from modalities across the increasingly diverse bioprocessing landscape.

## Graphical abstract

A platform approach to the affinity capture and enrichment of host cell proteins to enable their subsequent deep characterization and elucidation of variants or proteoforms (e.g., molecular size variants, charge variants, glycoforms, etc.).

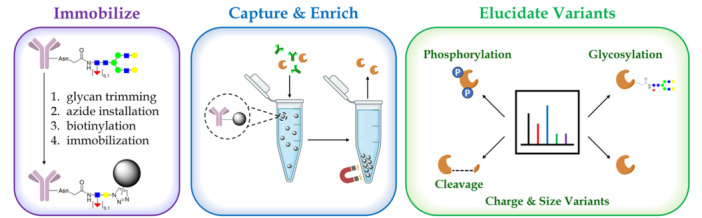

## 1 Introduction

Removing host cell protein (HCP) impurities is a principal goal of downstream processing for biotherapeutics, and purification processes routinely employ multiple orthogonal chromatography and filtration steps to promote their removal and to meet industry and regulatory expectations for residual HCP content (Gilgunn and Bones 2018; Y. Li 2017; Pezzini et al. 2011; Shukla and Hinckley 2008; Shukla et al. 2007). However, HCPs are a broad class of proteins, resulting in diverse physicochemical properties. Historically, a given HCP has generally been treated as a single molecular species with a single set of characteristics. On the contrary, as we demonstrate herein, multiple proteoforms or variants likely exist for a given HCP, further increasing their diverse physicochemical properties. By “proteoforms” or “variants” (which are commonly used synonymously), we refer to different molecular forms of a protein resulting from genetic variations, alternative splicing of RNA transcripts, and post‐translational modifications (PTMs) of the protein encoded by a given gene (Smith and Kelleher 2018; Smith and Kelleher 2013). Proteoforms can manifest as myriad glycoprotein variants, molecular size variants, and charge variants, among others. Notably, in addition to cellular processes, various chemical processes can generate variants during the production, storage, and purification of proteins, such as via reactive metabolites and light (Chumsae et al. 2013; Chumsae et al. 2015; Liu et al. 2014).

In recent years, advances in analytical techniques and instrumentation have enabled researchers to begin cataloguing numerous HCPs across hosts and biotherapeutic modalities. In the bioprocessing space, significant efforts have centered on Chinese hamster ovary (CHO) cells, a workhorse of recombinant therapeutic protein production and where increased scrutiny of HCPs initially took hold (Hamaker et al. 2022; Krawitz et al. 2006; Levy et al. 2014). These cataloguing efforts have borne substantial fruit, such as the identification of several enzymatic CHO HCPs (e.g., cathepsin D, lipoprotein lipase, and liposomal acid lipase, among others) which degrade either excipients in therapeutic protein formulations or therapeutic proteins themselves, even when present at sub‐parts per million (ppm) levels (Bee et al. 2015; Y. Chen et al. 2021; Chiu et al. 2017; Ranjan et al. 2019; Ranjan et al. 2019; Robert et al. 2009).

Despite significant progress in CHO HCP identification, these efforts remain hamstrung by analytical challenges, particularly the low abundance of HCPs: in a highly pure drug substance, the dynamic range between a therapeutic protein and its trace HCP contaminants may be 6 to 8 orders of magnitude (Graf et al. 2021). For mass spectrometry‐based methods, which are now the bedrock of HCP identification endeavors, the enormity of the therapeutic protein signal makes detection of these trace impurities extremely challenging. Some clever approaches have been devised to partially circumvent this problem, including therapeutic antibody depletion using Protein A affinity chromatography, enrichment of HCPs using molecular weight cutoff filters, enrichment of HCPs using ProteoMiner beads, and activity‐based profiling (I. H. Chen et al. 2020; I. H. Chen et al. 2020; Graf et al. 2021; R. O. Johnson et al. 2020; X. Li et al. 2021; S. S. Zhang et al. 2022). Novel peptide ligands conjugated to chromatography resins (e.g., LigaGuard^TM^, derived from research by the Menegatti and Carbonell groups) have also been developed for the targeted capture and removal of HCPs, where the capture and enrichment of the HCPs can aid in subsequent proteomic analysis (Lavoie et al. 2019; Lavoie et al. 2020; Reese et al. 2020; Sripada et al. 2022).

However, most efforts have focused primarily on HCP identification, and the success of these approaches has been case by case. More extensive exploration of the fundamental chemical nature of HCPs has not been undertaken. It is widely understood that, in principle, proteins expressed by mammalian cells exhibit a wide range of proteoforms or variants. For therapeutic proteins, these proteoforms—which can manifest as glycoprotein variants, molecular size variants, and charge variants, among others—are deeply and routinely investigated by analytical scientists. In contrast, while HCPs are endogenously expressed by the same hosts as our biotherapeutics, examination of their variants has received less attention, as evidenced by a dearth of such literature; the only analyses we found in the literature provided cursory examinations of (or suggested the potential for) N‐glycosylation and N‐terminal acetylation (Baycin‐Hizal et al. 2012; Wang et al. 2009). Because diversity in molecular architecture imparts biophysical diversity to the HCP proteoforms, a deeper understanding of the underlying protein chemistry of these impurities can lead to a deeper understanding of their interactions with other biomolecules, such as with our biotherapeutics. Shedding light on such interactions may help us better understand how such impurities copurify with biotherapeutics and, consequently, afford more tailored solutions to enable their removal during downstream processing.

The CHO HCP phospholipase B‐Like 2 (PLBL2, E.C. 3.1.1; UniProt G3I6T1) was first reported 10 years ago by Genentech and quickly gained notoriety for both potentially degrading polysorbate in the formulation of a sulfatase enzyme replacement therapy and for its propensity to copurify with therapeutic antibodies (Bateman et al. 2024; Dixit et al. 2016; Fischer et al. 2017; Martin Vanderlaan et al. 2015). Indeed, the drug substance of Genentech's lebrikizumab (a humanized monoclonal IgG4 antibody) suffered from residual CHO PLBL2 levels as high as ~330 ppm, which triggered immunogenic reactions in ~90% of subjects in a Phase III clinical trial for asthma. An overhaul of the downstream process for lebrikizumab was necessary to achieve CHO PLBL2 levels sufficiently low to enable subsequent trials (Fischer et al. 2017). More recent studies have demonstrated a lack of hydrolytic activity by CHO PLBL2 against polysorbates and their analogs, calling into question CHO PLBL2's putative role in polysorbate degradation (Bhargava et al. 2021; X. Li et al. 2021; S. Zhang et al. 2020). Nevertheless, CHO PLBL2 remains a persistent challenge for process engineers due to its propensity to copurify with therapeutic proteins and to cause immunogenic effects in patients. Surface plasmon resonance spectroscopy studies have demonstrated both preferential binding between CHO PLBL2 and IgG4 antibodies over other IgG isotypes and a potential multivalent binding mechanism, which may be driven by interactions with the antibody's complementarity‐determining regions (Tran et al. 2016). Therefore, CHO PLBL2 removal remains a special concern in the development of IgG4 antibodies or other therapies borrowing their molecular architecture.

Aside from this handful of studies, we have a dearth of knowledge of CHO PLBL2 and its biochemical characterization. CHO PLBL2 appears to be expressed as a ~ 66 kDa precursor, which may undergo limited proteolysis to achieve ~40 and ~28 kDa fragments (Martin Vanderlaan et al. 2015). But we know little about its glycosylation, phosphorylation, or other variants. To date, no reliable homology models or crystal structures have been published, and we still do not know CHO PLBL2's native substrate or role within typical CHO cell proliferation and subsistence.

Investigations of the mouse and human orthologs of PLBL2 provide some, albeit still limited, insight. The mouse ortholog is expressed as a ~ 75 kDa precursor, which likely undergoes limited proteolysis at multiple sites to render ~66, ~40, ~28, and ~15 kDa fragments, which remain associated through noncovalent interactions (Deuschl et al. 2006). Similarly, the human ortholog is expressed as a ~ 76 kDa precursor and undergoes proteolysis to afford ~45 and ~32 kDa fragments (Jensen et al. 2007). Both orthologs possess multiple sites exhibiting N‐glycosylation, including mannose 6‐phosphate, which is a hallmark of lysosomal matrix proteins and suggests that these species naturally reside in the lysosome (Deuschl et al. 2006; Jensen et al. 2007; Lakomek et al. 2009). Crystal structures of the mouse ortholog revealed a core αββα sandwich motif and N‐terminal cysteine residue in the ~40 kDa fragment, which is reminiscent of the N‐terminal nucleophile hydrolase superfamily (Lakomek et al. 2009). Nevertheless, there is still much to learn.

**Figure 1:**
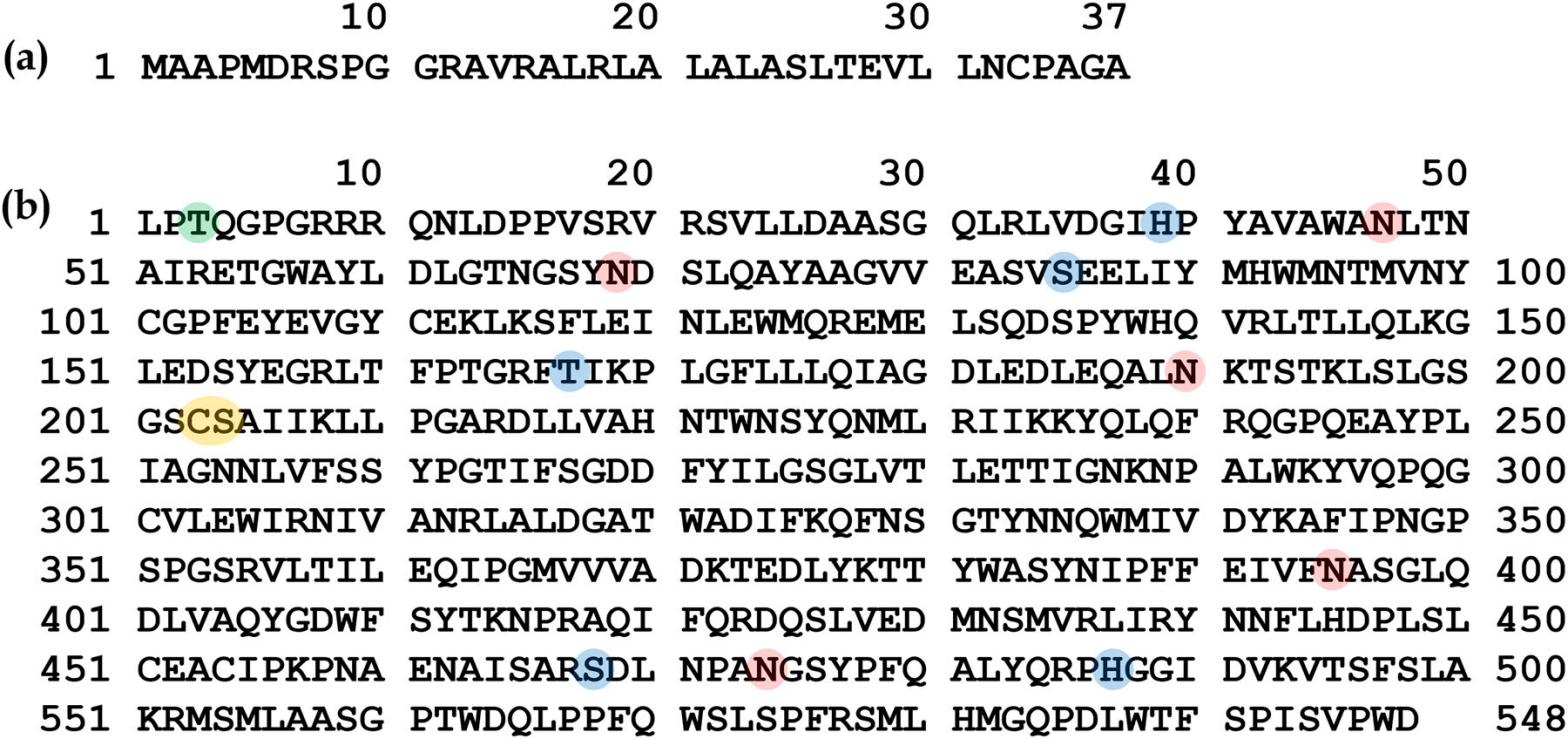
Amino acid sequence of CHO PLBL2, including (a) the signal sequence (which was not detected in the peptide mapping analysis) and (b) the main sequence (which was confirmed with a cumulative sequence coverage of 99% in the peptide mapping analysis), highlighting post‐translational modification sites identified: N‐linked glycosylation (red), O‐linked glycosylation (green), phosphorylation (blue), and cleavage (between C203 and S204, yellow).

To that end, we present—to the best of our knowledge—the first detailed characterization of a challenging CHO HCP recovered from a monoclonal antibody. Using CHO PLBL2 as a case study, we first employ site‐specifically modified and immobilized anti‐CHO PLBL2 antibodies for the targeted capture and isolation of CHO PLBL2 from pembrolizumab, which was stably expressed via CHO cells and copurified with pembrolizumab following a typical antibody purification process (i.e., harvest clarification, affinity capture, cation‐exchange chromatography, concentration, and formulation, where all expression and purification steps were performed by the vendor MedChemExpress). Our custom affinity resin successfully enriches the captured CHO PLBL2 by over 10‐fold, enabling mass spectrometry‐based and other characterization techniques to gather unprecedented understanding of CHO PLBL2's proteoforms, including charge variants, substantial N‐linked and O‐linked glycosylation, phosphorylation, and cleavage (including confident identification of the cleavage site) to render multiple molecular weight isoforms, as summarized in Figure 1.

Finally, efforts at identifying and removing challenging HCPs from non‐CHO host organisms for both therapeutic proteins and emerging modalities (e.g., adeno‐associated virus (AAV) and lentiviral vectors for gene therapy) are gaining momentum and increased scrutiny (Bracewell et al. 2021; S. Johnson et al. 2018; S. Zhang et al. 2024). While our work centered on CHO PLBL2, our affinity enrichment approach should be broadly applicable to enable the examination of other challenging HCPs from hosts and modalities across the increasingly diverse bioprocessing landscape.

## 2 Materials and Methods

### 2.1 Materials

Goat anti‐CHO PLBL2 polyclonal antibodies (GPLB‐65ALY), mouse anti‐CHO PLBL2 monoclonal antibodies (MPLB‐65ALY‐4D5), and recombinant CHO‐S PLBL2 (E.C. 3.1.1; UniProt G3I6T1; AG65‐0365‐Z) were from Immunology Consultants Laboratory (Portland, OR, USA). Pembrolizumab (HY‐P9902) was from MedChemExpress (Monmouth Junction, NJ, USA). As discussed in the introduction, MedChemExpress had stably expressed the pembrolizumab via CHO cells in a suspension culture and then purified the pembrolizumab via harvest clarification, affinity chromatography, cation‐exchange chromatography, and ultrafiltration/diafiltration to achieve a formulation of 100 mM proline‐acetate, 20 mM arginine (pH 5.0). Purified recombinant CHO phospholipase B‐like 2 (1582716) was from the United States Pharmacopoeia Convention (USP, Rockville, MD, USA). Protein concentrations were determined via UV–Vis absorption and extinction coefficients of 1.4 mL mg^−1^ cm^−1^ for the goat anti‐CHO PLBL2 polyclonal IgGs and mouse anti‐CHO PLBL2 monoclonal IgG, 2.0 mL mg^−1^ cm^−1^ for the CHO‐S PLBL2 (at 280 nm; supplied by the vendor), 1.41 mL mg^−1^ cm^−1^ for pembrolizumab (at 280 nm; calculated based on the amino acid sequence), and 1.915 mL mg^−1^ cm^−1^ for the USP CHO PLBL2 (at 280 nm; calculated based on the amino acid sequence). UV–Vis spectra for the goat anti‐CHO PLBL2 polyclonal IgGs, recombinant CHO‐S PLBL2, pembrolizumab, mouse anti‐CHO PLBL2 monoclonal IgG, and USP CHO PLBL2 are shown in Figure S.1 through Figure S.5, respectively.

The GlyCLICK® Azide Activation kit to modify up to 2 mg of IgG (L1‐AZ1‐200) was from Genovis AB (Cambridge, MA, USA and Lund, Sweden) and contained the following materials: immobilized GlycINATOR® (EndoS2; E.C. 3.2.1.96; UniProt T1WGN1) microspin column, β‐1,4‐galactosyltransferase (GalT Y289L; E.C. 2.4.1.22; UniProt P08037), UDP‐GalNAz, and 20X concentrate (0.5 M) of Tris‐buffered saline (TBS, pH 7.4).

DBCO‐PEG_4_‐biotin (CCT‐A105‐5) and high‐capacity streptavidin beads (CCT‐1497‐5) were from Vector Laboratories (Newark, CA, USA). Concentrations of DBCO‐PEG_4_‐biotin samples were determined via UV–Vis absorption and extinction coefficient of 20,000 M^−1^ cm^−1^ (at 307 nm; supplied by the vendor). The structure and UV–Vis spectra for DBCO‐PEG_4_‐biotin are shown in Figure S.6 through Figure S.7, respectively.

The protein concentrator (0.5 mL, 30 kDa MWCO; 88502), DynaMag^TM^‐2 magnet (12321D), bovine serum albumin (BSA; 23209), Novex^TM^ IEF protein gel (pH 3–7; EC66452BOX), Novex^TM^ IEF anode buffer (50X concentrate; LC5300), Novex^TM^ IEF cathode buffer (pH 3–7; 10X concentrate; LC5370), Novex^TM^ IEF sample buffer (pH 3–7; 2X concentrate; LC5371), Invitrolon^TM^ PVDF Filter Paper Sandwich (LC2005), Xcell SureLock® Mini‐Cell (EI0001), and Novex XCell II^TM^ blot module (EI9051) were from Thermo Fisher Scientific (Waltham, MA, USA). Protein concentrations for the BSA were determined via UV–Vis absorption using an extinction coefficient of 0.67 mL mg^−1^ cm^−1^ (at 280 nm; supplied by the vendor); the UV–Vis spectra are shown in Figure S.8. Precision Plus Protein^TM^ standard (1610373) was purchased from Bio‐Rad Laboratories (Hercules, CA, USA). One‐Step Blue® protein gel stain (21003‐1L) was from Biotium (Fremont, CA, USA).

The hamster PLBL2 enzyme‐linked immunosorbent assay (ELISA) kit (MBS564202) was from MyBioSource (San Diego, CA, USA) and included the following components: antibody‐coated ELISA micro plate, detection antibody (100× concentrate), HRP‐conjugated streptavidin (100× concentrate), calibrator, diluent solution, wash solution (20× concentrate), chromogen‐substrate solution (3,3′,5,5′‐ tetramethylbenzidine dihydrochloride, TMB), and stop solution (0.3 M sulfuric acid). The CHO PLBL2 ELISA assay control was purified CHO‐3E7 PLBL2 (AG65‐0324) from Immunology Consultants Laboratory, diluted to a concentration 300 ng/mL. The diluent solution was from the hamster PLBL2 ELISA kit.

Tris base (4109‐02), hydrochloric acid (5619‐02), glacial acetic acid (9522‐05), sodium hydroxide (5000‐03), polysorbate 80 (4125‐04), and polysorbate 20 (4116‐04) were from JT Baker (part of Avantor; Radnor, PA, USA). Formic acid (94318‐250 ML) was from Honeywell (Charlotte, NC, USA).

The Criterion^TM^ PowerPac Universal power supply (1656019) was from Bio‐Rad Laboratories (Hercules, CA, USA). IEF fixing solution (2× concentrate; F7264) was from Sigma‐Aldrich (St. Louis, MO, USA). IEF marker (pH 3–10; 39212.01) was from Serva Electrophoresis GmbH (Heidelberg, Germany). Methanol (A935‐4) was from Fisher Scientific (Waltham, MA, USA). Donkey anti‐mouse (H&L) polyclonal antibody peroxidase conjugated (610‐703‐002) was from Rockland Immunochemicals (Pottstown, PA, USA). ECL^TM^ Western blot analysis detection reagents (RPN2106) were from Cytiva (Marlborough, MA, USA). Disposable square petri dishes (25378‐045) were from VWR (part of Avantor; Radnor, PA, USA). Dulbecco's phosphate‐buffered saline (10× Concentrate; 14200‐075) was from Gibco Waltham, MA, USA. Dry milk (M0841) was from Lab Scientific bioKEMIX (Danvers, MA, USA). Formic acid (94318‐250 ML) was from Honeywell (Charlotte, NC, USA).

The 12–230 kDa Wes separation module (SM‐W004) was from Protein Simple (San Jose, CA, USA) and contained the following materials: capillary cartridges (each containing 25 capillaries); 12–230 kDa prefilled plates; 10× sample buffer; wash buffer; and lyophilized DTT, fluorescent master mix, and biotinylated ladder. The anti‐mouse detection module (DM‐002) was also from Protein Simple and contained the following materials: anti‐mouse HRP conjugate secondary antibody, Luminol‐S, peroxide, and antibody diluent.

S‐Trap micro spin columns were from ProtiFi (Fairport, NY, USA); dithiothreitol (DTT, 1 M solution), iodoacetamide (IAM, single‐use, pre‐weighed 9.3 mg), tris(hydroxymethyl)aminomethane (Tris, 1.0 M buffer solution., pH 7.5), and tetraethylammonium bicarbonate (TEAB, 1.0 M, pH 8.5 ± 0.1) were from Sigma‐Aldrich (St. Louis, MO, USA); phosphoric acid (o‐phosphoric acid, 85%, Certified ACS) and methanol were from Fisher Chemical (Waltham, MA, USA); formic acid (LC‐MS grade), acetonitrile (LC‐MS grade, 99.8%), and sodium dodecyl sulfate (SDS, 20% solution) were from Thermo Fisher Scientific (Waltham, MA, USA); LC‐MS grade water was from Honeywell (Charlotte, NC, USA); and trypsin (Trypsin Platinum, mass spectrometry grade) was from Promega (Madison, WI, USA).

### 2.2 Site‐Specific Modification and Immobilization of Goat Anti‐CHO PLBL2 Polyclonal IgG

#### 2.2.1 Chemo‐Enzymatic, Site‐Specific Modification

The site‐specific modification of 2 mg of goat anti‐CHO PLBL2 polyclonal IgGs (which includes buffer exchange, Fc N‐glycan cleavage by EndoS2, and azide activation via GalT) followed the procedure previously reported by our group (Dolan et al. 2024), rendering 1.3 mg of azide‐activated IgG.

#### 2.2.2 Site‐Specific Conjugation of Azide‐Activated IgG to DBCO‐PEG_4_‐Biotin

DBCO‐PEG_4_‐biotin (5 mg, 6.7 µmol) was reconstituted using 670 µL of water to a final concentration of 10 mM. About 1.3 mg of azide‐activated anti‐CHO PLBL2 IgG was mixed with 10 mM DBCO‐PEG_4_‐biotin at a molar ratio of 50:1 (DBCO‐PEG_4_‐biotin:azide) and IgG concentration of 1.0 mg/mL (or 6.7 µM) in 1× TBS (pH 7.4). The reaction contents were incubated using a MultiTherm heating/cooling shaker for 22 h at 25°C while agitating at 1000 rpm and shielding from light. Excess DBCO‐PEG_4_‐biotin was removed after conjugation following the buffer‐exchange procedure described previously (Dolan et al. 2024). About 1.1 mg of IgG (at 3.1 mg/mL or 7.3 µM) were recovered for a yield of 82%.

#### 2.2.3 Immobilization of Biotinylated IgG to Streptavidin Magnetic Beads

High‐capacity streptavidin magnetic beads were prepared for and subsequently used in the immobilization of the biotinylated anti‐CHO PLBL2 IgGs, as described in the Supporting Information. About 1.1 mg of IgG was successfully immobilized.

### 2.3 Affinity Capture of Endogenous CHO PLBL2 From Pembrolizumab (IgG4) and Enrichment

A 50 mg vial of pembrolizumab (formulated in 100 mM proline‐acetate and 20 mM arginine at pH 5.0 and at a protein concentration of 7.29 mg/mL, or 49.7 µM) was removed from storage at ≤−65°C and thawed at room temperature. To prepare for the affinity capture, the pembrolizumab was adjusted to pH 7.0 using 1 M Tris (pH 8.0), rendering a solution with a final concentration of 6.2 mg/mL (42 µM).

The capture of endogenous CHO PLBL2 proceeded by incubating the immobilized goat IgG with pembrolizumab, such that the CHO PLBL2 was at sub‐saturating conditions: 250 µg (1.66 nmol in 250 µL of bead solution) of immobilized goat IgG were mixed with 54 µg (0.8 nmol) of CHO PLBL2 to achieve a molar ratio of 0.5:1 (CHO PLBL2:IgG), assuming an endogenous CHO PLBL2 concentration of 8 µg/mL based on previous studies. These conditions were informed by batch uptake experiments to determine the static binding capacity (about 0.01 gram of CHO PLBL2 per gram of custom resin at 10% breakthrough; data not shown). The solution was mixed end over end for 19.5 h at 2°C–8°C while shielding from light.

Following incubation, 1 mL of the solution was added to a clean 1.5 mL Eppendorf tube, which was placed onto the DynaMag^TM^‐2 to magnetize and separate the beads from the solution. The supernatant from the Eppendorf tube was collected into a separate 15 mL conical tube and replaced with another 1 mL of affinity capture solution. This process was repeated until all supernatant from the incubation had been separated from the beads and collected. The beads were then washed using 5 BVs (i.e., 5 × 250 µL) of 50 mM Tris (pH 8.0), 3 BVs of 50 mM Tris with 1% (w/v) polysorbate 80 (pH 9.0), and 3 BVs of 50 mM Tris (pH 8.0). Bound CHO PLBL2 was stripped from the immobilized anti‐CHO PLBL2 IgG using 3×80 µL (~1/3 BV) of 1% (w/v) formic acid (pH 2.2).

## 3 CHO PLBL2 ELISA

The CHO PLBL2 ELISA was performed as described in the Supporting Information.

### 3.1 Analysis of Molecular Size Isoforms via Capillary Western Blot

The CHO PLBL2 content of the pembrolizumab starting material (i.e., load), pembrolizumab after affinity capture (i.e., depleted of CHO PLBL2), and strip (or enriched) fractions (i.e., enriched and recovered CHO PLBL2) was analyzed via capillary Western blot, as described in the Supporting Information.

### 3.2 Analysis of Charge Isoforms via Isoelectric Focusing (IEF) Gel and Western Blot

Analysis of charge isoforms via IEF gel and Western blot was performed as described in the Supporting Information.

### 3.3 Peptide Mapping Analysis

The preparation of samples for peptide mapping analysis was performed as described in the Supporting Information. The five samples include: (1) Load: pembrolizumab starting material, (2) Depleted: pembrolizumab after custom affinity capture (i.e., depleted of endogenous CHO PLBL2), (3) Enriched: pool of enriched fractions (i.e., enriched and recovered endogenous CHO PLBL2), (4) USP: USP recombinant CHO PLBL2 (Catalog #1582716, Lot #F174K0), and (5) iCLlab: Immunology Consultants Laboratory recombinant CHO‐S PLBL2 (Catalog #AG65‐0365Z, Lot #5).

For LC‐MS analysis, 30 µL of each digest (containing 15 µg of digested total protein) was analyzed using an ACQUITY UPLC I‐Class PLUS System (Waters, Milford, MA, USA) coupled to an Orbitrap Exploris 240 mass spectrometer (Thermo Fisher Scientific, Waltham, MA, USA). The LC‐MS system was controlled with Xcalibur 4.6 software (Thermo Fisher Scientific, Waltham, MA, USA). Before analysis, digests were stored in an autosampler set at 4°C. The sample loop was set offline for 2 min. The autosampler washing program included a weak wash with 2000 µL of water and a strong wash with 1000 µL of 80% acetonitrile in water. Reverse‐phase chromatographic separation was performed on an ACQUITY UPLC Peptide CSH C18 Column (130 Å, 1.7 µm, 2.1 mm × 150 mm) at 40°C. The mobile phase used a complex gradient of Solvent A (0.1% (w/v) formic acid in water) and Solvent B (0.1% (w/v) formic acid in acetonitrile), starting at 1% B (maintained for 5 min), increasing to 40% B over 85 min, and reaching 95% B at 100 min, with a flow rate of 200 µL/min. After reaching 95% B, the system underwent three alternating organic flush cycles (95% B to 5% B to 95% B), followed by column re‐equilibration at 1% B at a flow rate of 300 µL/min. UV detection was set at 214 nm.

The Orbitrap Exploris 240 mass spectrometer was operated with an H‐ESI ion source using specific conditions: spray voltage of 3500 V (positive ions), ion transfer tube temperature of 320°C, vaporizer temperature of 275°C, and sheath and auxiliary gas flows at 35 and 7 Arb, respectively. MS1 scans in the data‐dependent acquisition (DDA) mode were performed over a mass range of 195–2900 m/z with an Orbitrap resolution of 60,000. The RF lens was set at 70%, AGC target at 300% (normalized), and maximum injection time at 100 ms. Data were collected in profile mode under positive polarity without source fragmentation, using EASY‐IC for internal mass calibration. DDA MS2 settings included monoisotopic peak determination set to “Peptide” with relaxed restrictions when few precursors were found, intensity threshold at 1.0e4, and charge states 2–5 included (undetermined charge states excluded). Dynamic exclusion was set to exclude ions after one MS2 event, with a 3‐second exclusion duration and ±3 ppm mass tolerance. Isotopes were excluded from analysis, and up to 10 data‐dependent scans were allowed per duty cycle.

Peptide mapping data were processed using Byos version 5.5, part of the PMI software package (Protein Metrics by Dotmatics; Boston, MA, USA), with manual verification of each identified peptide. Briefly, raw LC‐MS/MS data files, together with the PLBL2 protein sequence (UniProt entry G3I6T1) and pembrolizumab heavy chain and light chain sequences (from Protein Data Bank entry 5DK3) (Scapin et al. 2015) were loaded as input to the “PTM” workflow in Byos for peptide‐spectrum matching using the Byonic algorithm. Most Byonic parameters were set to default values, with minor adjustments as follows: digestion cleavage sites were specified as “RK” with C‐terminal cleavage specificity, full specificity was enforced, and no missed cleavages were allowed. Fixed modifications included carbamidomethylation (+57.021464) at C and Gln→pyro‐Glu (−17.026549) at protein N‐terminal Q, while rare modifications allowed at a maximum of one occurrence included (De)Gln→pyro‐Glu (+17.026549 at protein N‐terminal Q), phosphorylation (+79.966331 at H, S, T, Y), and 6x His‐tag (+822.353472 at protein C‐terminal). Both total common and total rare modifications were capped at one. The glycan search included Byos database files: “N‐glycan 309 mammalian no sodium.txt” and “O‐glycans 78 mammalian.txt.” Identified peptides were manually reviewed to eliminate mis‐annotations and adjust retention time/chromatogram integration windows. Finally, peptide identifications were exported as an “in silico list” with appropriate charge states and elution windows for relative quantitation of glycopeptides using the “PTM Pure In‐Silico” workflow. The intensity for peptide quantitation was calculated as the sum of integrated extracted chromatographic peak areas of the top charge states, selected based on consistency across glycoforms of each peptide sequence. The signature peptides LSLGSGSC and SAIIK, corresponding to the truncation site between C203 and S204, were further verified by manually extracting chromatograms of their identified charge states using FreeStyle 1.8 software (Thermo Fisher Scientific, Waltham, MA, USA).

### 3.4 Homology Model Prediction and Structural Analysis

The structure of intact CHO PLBL2 was predicted via AlphaFold2 using the amino acid sequence from UniProt entry G3I6T1 (Jumper et al. 2021). pLDDT values for individual residues were averaged to determine the global pLDDT value for the predicted structure. Following structure prediction, PyMOL (version 3.1.4.1) was used to generate a structure of intact CHO PLBL2 with the signaling sequence (corresponding to the first 37 residues of UniProt entry G3I6T1, as seen in Figure 1) removed. In addition, structures of truncated CHO PLBL2 were generated by removing residues L1 through C203 (rendering the C‐terminal peptide, as described in the Results and Discussion section) or by removing residues S204 through D548 (rendering the N‐terminal peptide). To interrogate conformational changes associated with truncation, the N‐terminal peptide and C‐terminal peptide structures were imported into the Molecular Operating Environment (version 2020. 0901) and subjected to the QuickPrep procedure (using default parameters) for minor structure and charge state corrections, followed by energy minimization using the Amber10 forcefield (using default parameters). The convergence criterion for energy minimization was set based on the root mean square gradient of 0.001 kcal/mol/Å.

## 4 Results and Discussion

### 4.1 CHO PLBL2 Is Successfully Captured from Pembrolizumab and Subsequently Enriched, Enabling In‐Depth Characterization

**Scheme 1:**
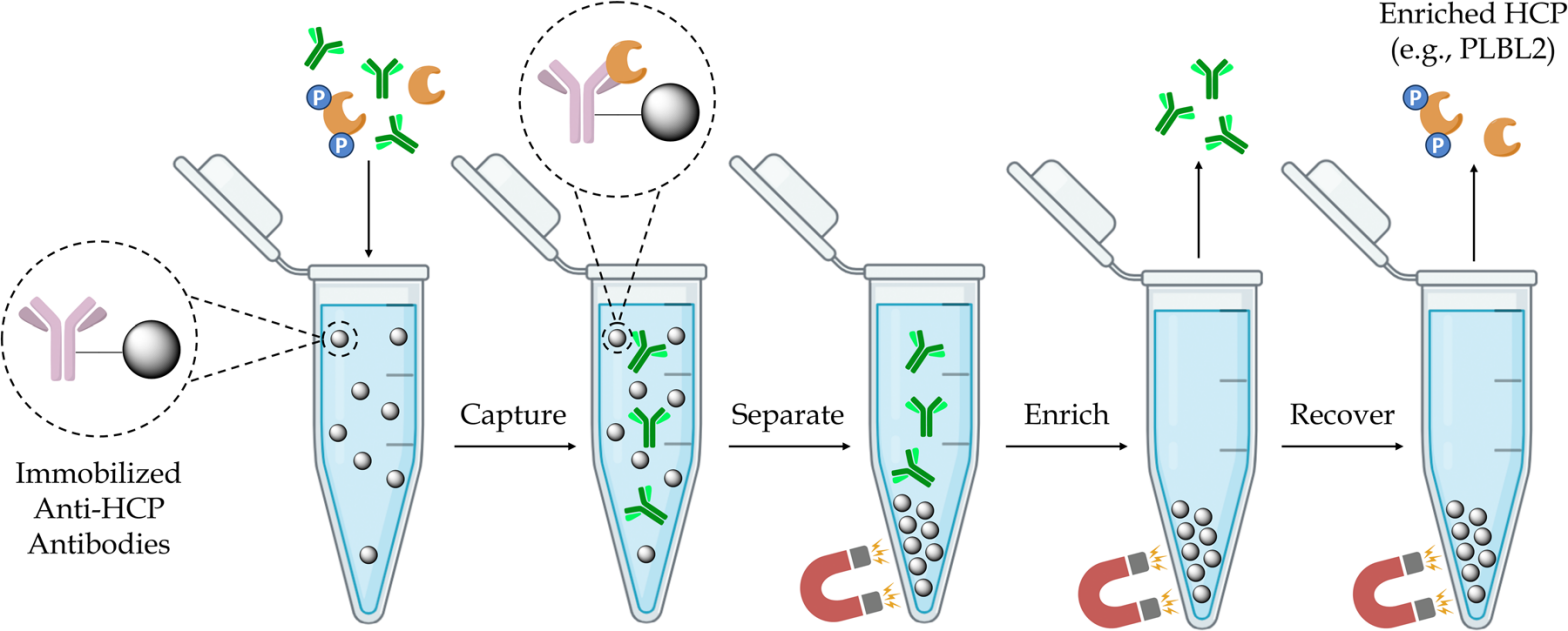
Affinity capture of a target host cell protein and its various proteoforms (e.g., CHO PLBL2, shown in orange) from a therapeutic antibody (e.g., pembrolizumab, an IgG4) using immobilized polyclonal antibodies directed against the HCP of interest, followed by enrichment and recovery of the target HCP and its proteoforms from the resin for subsequent characterization.

As depicted in Scheme 1, the present work showcases the capture and enrichment of CHO PLBL2 (which was endogenously expressed alongside pembrolizumab) using a custom affinity resin comprised of site‐specifically modified and immobilized goat polyclonal anti‐CHO PLBL2 IgGs (Dolan et al. 2024). Because of the expected diversity of the CHO PLBL2 variants, a chromatography resin that recognizes and binds to numerous epitopes was considered most suited to effectively capture, isolate, and enrich CHO PLBL2 from the pembrolizumab mixture. The pembrolizumab (purchased from MedChemExpress) exhibited >520 ppm of residual CHO PLBL2, even after undergoing extensive purification via harvest clarification, affinity chromatography, cation‐exchange chromatography, and ultrafiltration/diafiltration. Nevertheless, after washing the beads, the depleted pembrolizumab possessed only 0.7 ppm of CHO PLBL2 (as confirmed by CHO PLBL2 ELISA) at >85% yield, even without substantial optimization. The enriched CHO PLBL2 was then recovered by disrupting the antigen‐antibody interaction by exposure to 1% formic acid (pH 2.2). The material enriched in CHO PLBL2 was recovered as three fractions, which were analyzed as distinct fractions in the capillary Western blot and the IEF gel coupled to a traditional Western blot but were pooled together for peptide mapping.

Our custom affinity resin and capture workflow achieved a 10‐fold enrichment of CHO PLBL2, enabling enhanced detection by subsequent analyses, including boosting sequence coverage in peptide mapping analysis from approximately 60% to 85%. Critically, PTM sites that were completely undetectable in the load material (where no peptide‐spectrum matches were observed due to the suppressive effects of glycosylation on mass spectrometric detectability) became readily identifiable in the enriched material (Han and Costello 2013). Without referencing recombinant CHO PLBL2 standards, we confidently assigned multiple glycosylation sites: O‐glycosylation on T3 (2 glycoforms) and N‐glycosylation on N47 (4 glycoforms), N190 (1 glycoform), N395 (2 glycoforms), and N474 (2 glycoforms). Furthermore, by incorporating recombinant CHO‐S PLBL2 standards, we achieved the identification of even more glycoforms (Table 1), highlighting the combined power of affinity enrichment and complementary reference data in overcoming analytical challenges posed by PTMs. Overall, this methodology enabled deeper characterization of CHO PLBL2's proteoforms, especially glycoforms.

**Table 1:** Summary of N‐glycosylation, O‐glycosylation, and phosphorylation identified for endogenous CHO PLBL2.

Modification	Site	Total site occupancy (%)	Total glycoforms identified
N‐Linked Glycosylation	N47	99	17
	N69	100	6
	N190	99	46
	N395	99	56
	N474	100	41
O‐Linked Glycosylation	T3	94	6
Phosphohistidine	H39	23	N/A
	H487	2	
Phosphoserine	S85	52	N/A
	S468	2	
Phosphothreonine	T167	20	N/A

### 4.2 CHO PLBL2 Exhibits Numerous Charge Isoforms

Although recognized in principle that eukaryotic expression systems such as CHO cells introduce heterogeneity into their expressed proteins via post‐translational and other modifications, such proteoforms or variants have not been convincingly demonstrated for CHO HCPs, including PLBL2.

**Figure 2:**
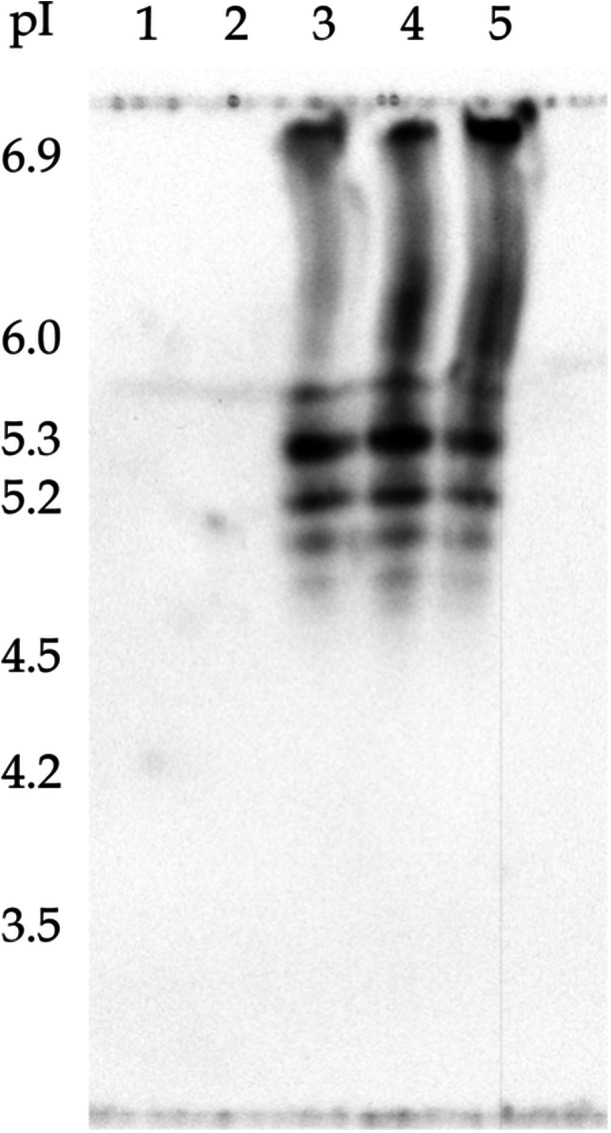
Analysis via IEF gel and Western blot of the charge variants of CHO PLBL2 captured from pembrolizumab. Lanes 1–2: isoelectric point (pI) markers. Lanes 3–5: enriched CHO PLBL2. Numerous distinct protein bands are observed between pIs of about 5.0 and about 6.0, indicating a diverse array of charge isoforms for CHO PLBL2 within this range. Bands are also observed at pIs > 6.9, which were not fully resolved. For reference, the theoretical pI for CHO PLBL2 (calculated based on the amino acid sequence of UniProt entry G3I6T1) is 5.63.

Analysis via isoelectric focusing (IEF) gel electrophoresis followed by a traditional Western blot revealed that CHO PLBL2 exhibits numerous and distinct charge isoforms. As seen in Lanes 3 through 5 of Figure 2 (and Figure S.9 in the Supporting Information), CHO PLBL2 captured from pembrolizumab demonstrates several distinct protein bands between isoelectric points (pI) of about 5.0 and about 6.0, indicating a diverse array of charge isoforms within this pI range. For reference, the theoretical pI for CHO PLBL2 (calculated based on the amino acid sequence of UniProt entry G3I6T1) is 5.63 (Martin Vanderlaan et al. 2015). Bands are also observed at pIs > 6.9, although these were not fully resolved on the IEF gel or in the Western blot.

### 4.3 CHO PLBL2 Also Exhibits Numerous Molecular Size Isoforms

**Figure 3:**
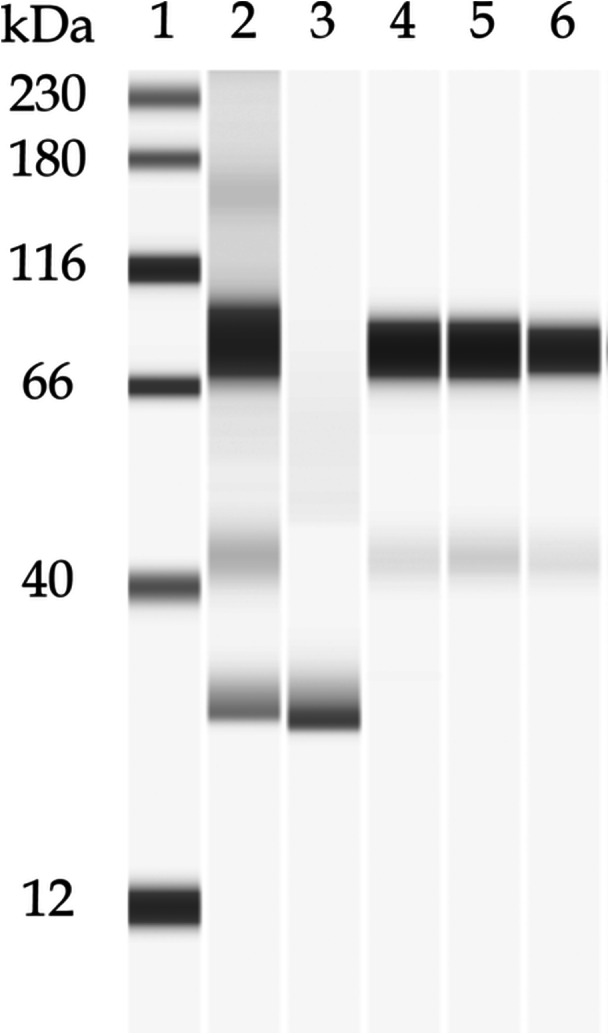
Affinity capture of CHO PLBL2 from pembrolizumab and subsequent enrichment, as detected via capillary Western blot. Lane 1: molecular weight marker. Lane 2: pembrolizumab starting material (pre‐enrichment, “load”), which exhibits multiple molecular weight isoforms (~80 kDa, ~45 kDa, and ~30 kDa) of endogenous CHO PLBL2. Lane 3: pembrolizumab after affinity capture (“depleted”), which exhibits depletion of the ~80 kDa and ~45 kDa isoforms of endogenous CHO PLBL2 but retention of the ~30 kDa isoform. Lanes 4–6: enriched fractions, which exhibit recovery and enrichment of primarily the ~80 kDa and ~45 kDa isoforms of endogenous CHO PLBL2.

CHO PLBL2 was also found to exhibit several molecular size isoforms. As seen in Figure 3 and Figure S.10 in the Supporting Information, the pembrolizumab starting material (“load”), pembrolizumab after affinity capture and depletion of endogenous CHO PLBL2 (“depleted”), and fractions that contained enriched and recovered endogenous CHO PLBL2 (“enriched”) were analyzed via capillary Western blot.

As seen in Lane 2 of Figure 3 and in Figure S.10a, CHO PLBL2 in the pembrolizumab starting material exhibits three molecular size isoforms, including a major isoform which migrates to ~80 kDa and other isoforms which migrate to ~45 and ~30 kDa. As seen in Lane 3 of Figure 3 and Figure S.10a, affinity capture of the endogenous CHO PLBL2 by the custom affinity resin depletes pembrolizumab of the CHO PLBL2 isoforms that migrate to ~80 and ~45 kDa. Interestingly, the isoform that migrates to ~30 kDa may not be bound by the custom affinity resin to a significant degree, as it is still observed in the depleted pembrolizumab. These observations again highlight the challenges in the separation of HCPs. Finally, the enriched material exhibits isoforms which migrate to ~80 and ~45 kDa (Lanes 4–6 of Figure 3); a small peak corresponding to the isoform which migrates to ~30 kDa is observed in the electropherogram in Figure S.10b.

As discussed in the introduction, literature for the mouse and human orthologs of CHO PLBL2 suggests that these molecular size variants may arise from limited proteolysis of PLBL2 following translation (Deuschl et al. 2006; Jensen et al. 2007; Martin Vanderlaan et al. 2015). Initial attempts to characterize these variants using intact mass analysis were unsuccessful due to poor signal intensity, likely attributed to extensive glycosylation. Additionally, the highly heterogeneous mass spectra could not be effectively deconvoluted to resolve distinct mass variants. This outcome is consistent with the presence of complex N‐glycosylation, O‐glycosylation, and phosphorylation. To further investigate these molecular size variants, we relied on peptide mapping analysis to identify potential cleavage sites.

### 4.4 Identification of CHO PLBL2 Cleavage Sites to Render Molecular Size Isoforms

**Figure 4:**
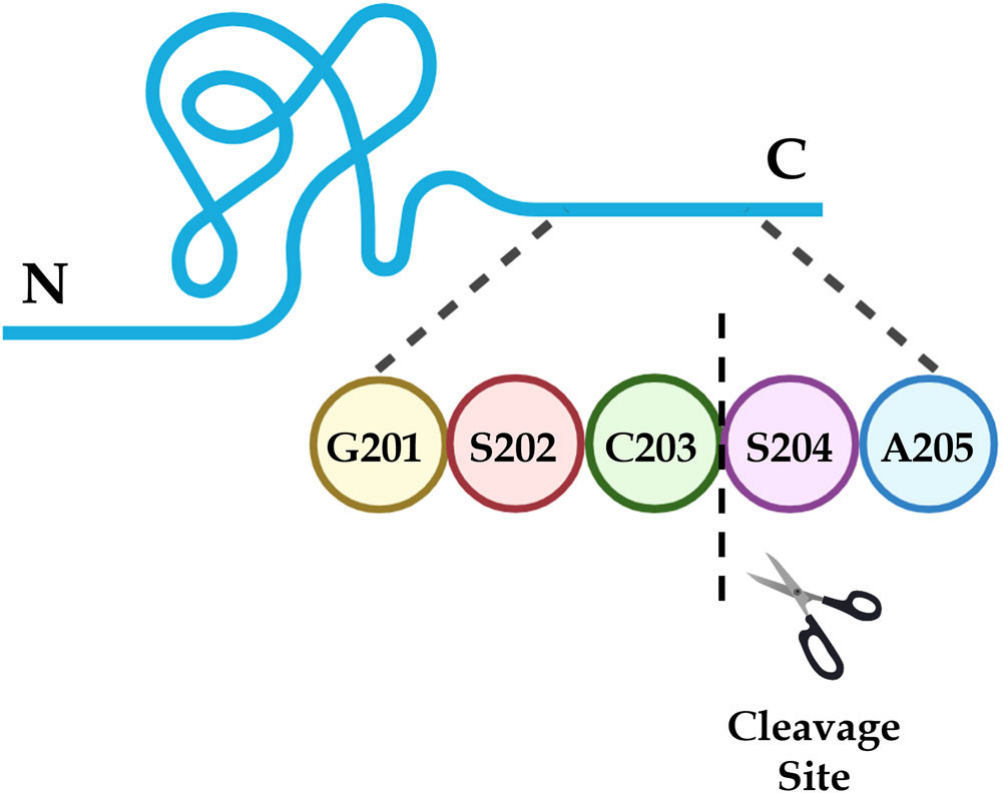
Cleavage site between C203 and S204, identified by peptide mapping in CHO PLBL2 captured from pembrolizumab. Truncation at this site yields an N‐terminal peptide of approximately 28 kDa and a C‐terminal peptide of approximately 44 kDa.

As depicted in Figure 4, we successfully located the truncation site between C203 and S204. Peptide mapping analysis of the enriched material identified tryptic peptides LSLGSGSC and SAIIK with high mass accuracy, indicating cleavage between C203 and S204. The peptide LSLGSGSC was observed as [M + H]+ at *m/z* 780.3557 (LC retention time: 18.0 min), closely matching its theoretical *m/z* of 780.3556 at a mass error of +0.1 ppm. Similarly, SAIIK was detected as [M + 2H]++ at *m/z* 266.1787, with LC retention times of 11.4 min and 12.3 min, at a mass error of 0.0 ppm. The presence of two retention times for SAIIK suggests the existence of stereoisomers. The full‐length peptide, LSLGSGSCSAIIK, was also observed as [M + 2H]++ at *m/z* 646.8475 (LC retention time: 32.3 min), closely matching its theoretical *m/z* of 646.8476 at a mass error of −0.2 ppm.

**Figure 5:**
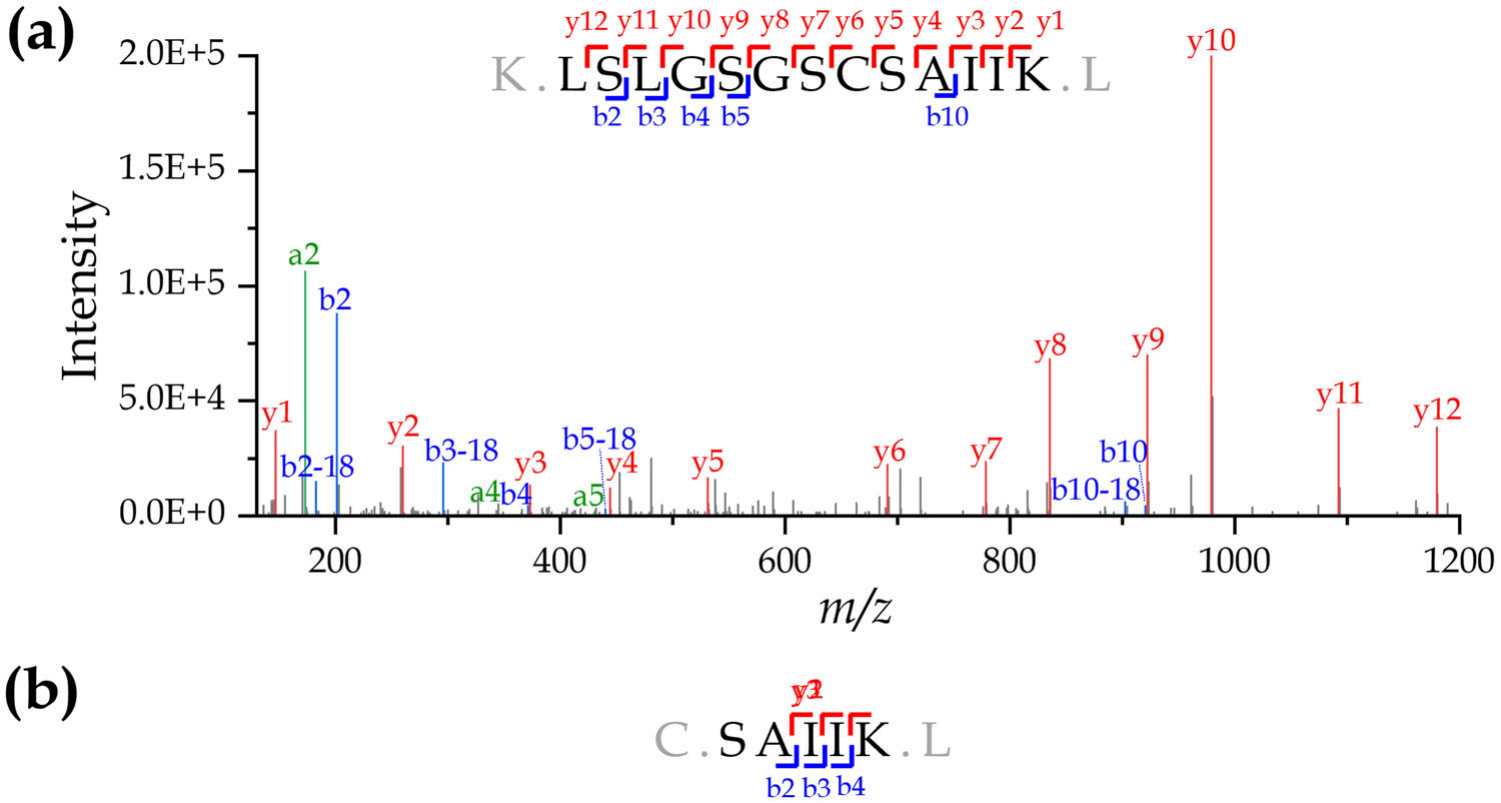
Tandem mass spectrum sequencing evidence confirming the cleavage site between C203 and S204 in CHO PLBL2 captured from pembrolizumab. Peptide mapping analysis generated high‐quality tandem mass spectra for (a) the full‐length tryptic peptide LSLGSGSC*SAIIK and (b) the C‐terminal tryptic peptide **SAIIK**, which resulted from C203/S204 cleavage. Note: As described in the Materials and Methods, cysteine was alkylated with iodoacetamide, resulting in carbamidomethylation (+57 Da).

The corresponding tandem mass spectra (MS2) of LSLGSGSCSAIIK and SAIIK are shown in Figure 5. However, the MS2 spectrum of LSLGSGSC [M + H]+ was not captured due to charge exclusion settings in the LC‐MS/MS method. The differences in observed retention times have ruled out potential artifacts from in‐source fragmentation of the full‐length peptide, further confirming that the truncation site between C203 and S204 resulted from limited proteolysis of CHO PLBL2 following translation.

Taken together, such cleavage would yield an N‐terminal peptide of ~28 kDa and C‐terminal peptide of ~44 kDa, which align well with those determined by the capillary Western blot analysis.

**Figure 6:**
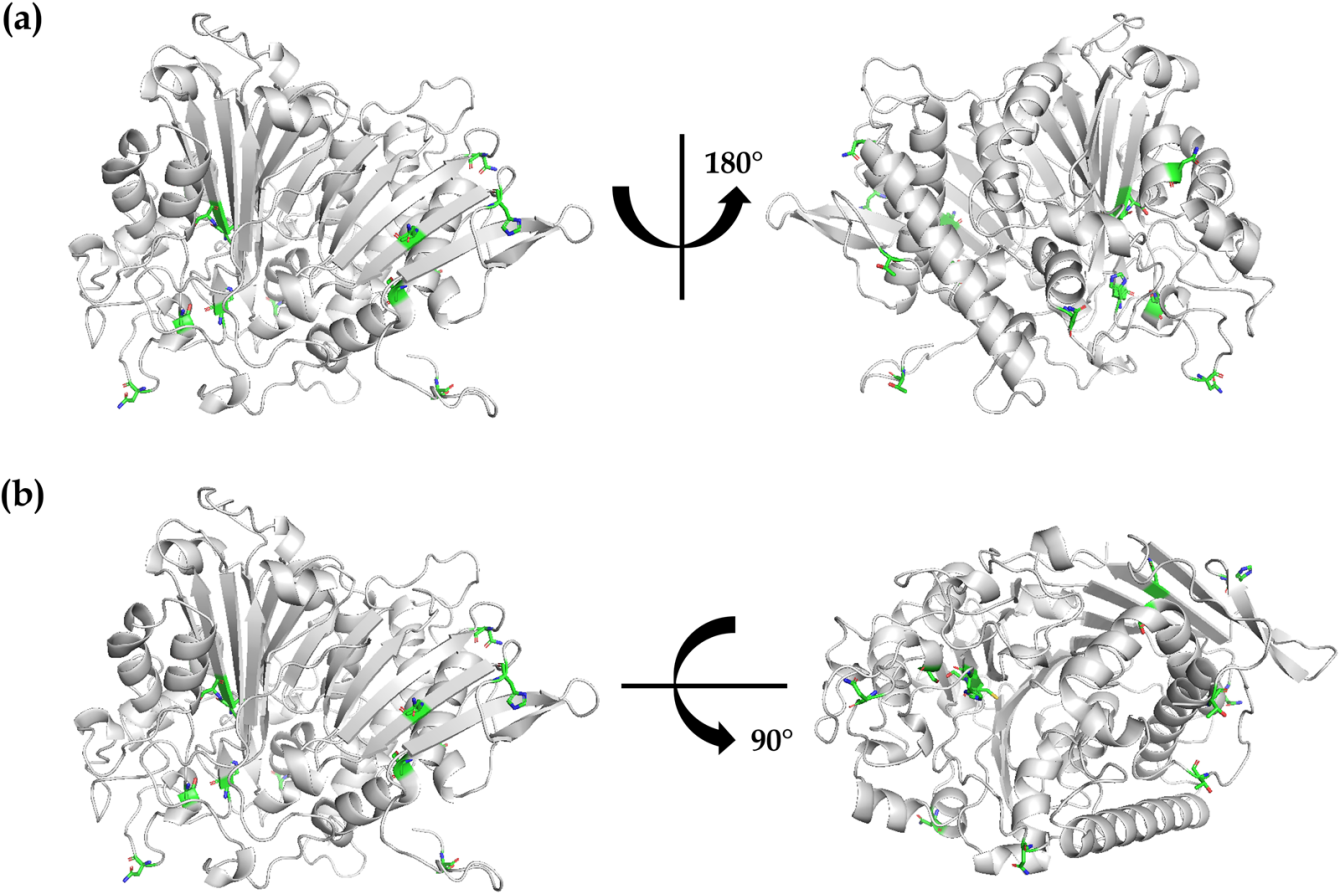
Structural analysis of intact CHO PLBL2 using a homology model predicted by AlphaFold2 using UniProt entry G3I6T1 (with the signaling sequence removed). Shown in color are residues which exhibit post‐translational modifications (including N‐linked glycosylation, O‐linked glycosylation, and phosphorylation) and the cleavage site to render N‐terminal and C‐terminal peptides. (a) Front and back views of intact CHO PLBL2. (b) Front and top views of intact CHO PLBL2.

As seen in Figure 6, the structure of intact CHO PLBL2 was predicted via AlphaFold2 using the amino acid sequence from UniProt entry G3I6T1 (Jumper et al. 2021), which possessed a global pLDDT score of 91.29. Notably, individual pLDDT scores for the first 51 residues were < 70. Therefore, following structure prediction, PyMOL (version 3.1.4.1) was used to generate a structure of intact CHO PLBL2 with the signaling sequence (corresponding to the first 37 residues of UniProt entry G3I6T1, as seen in Figure 1a) removed.

**Figure 7:**
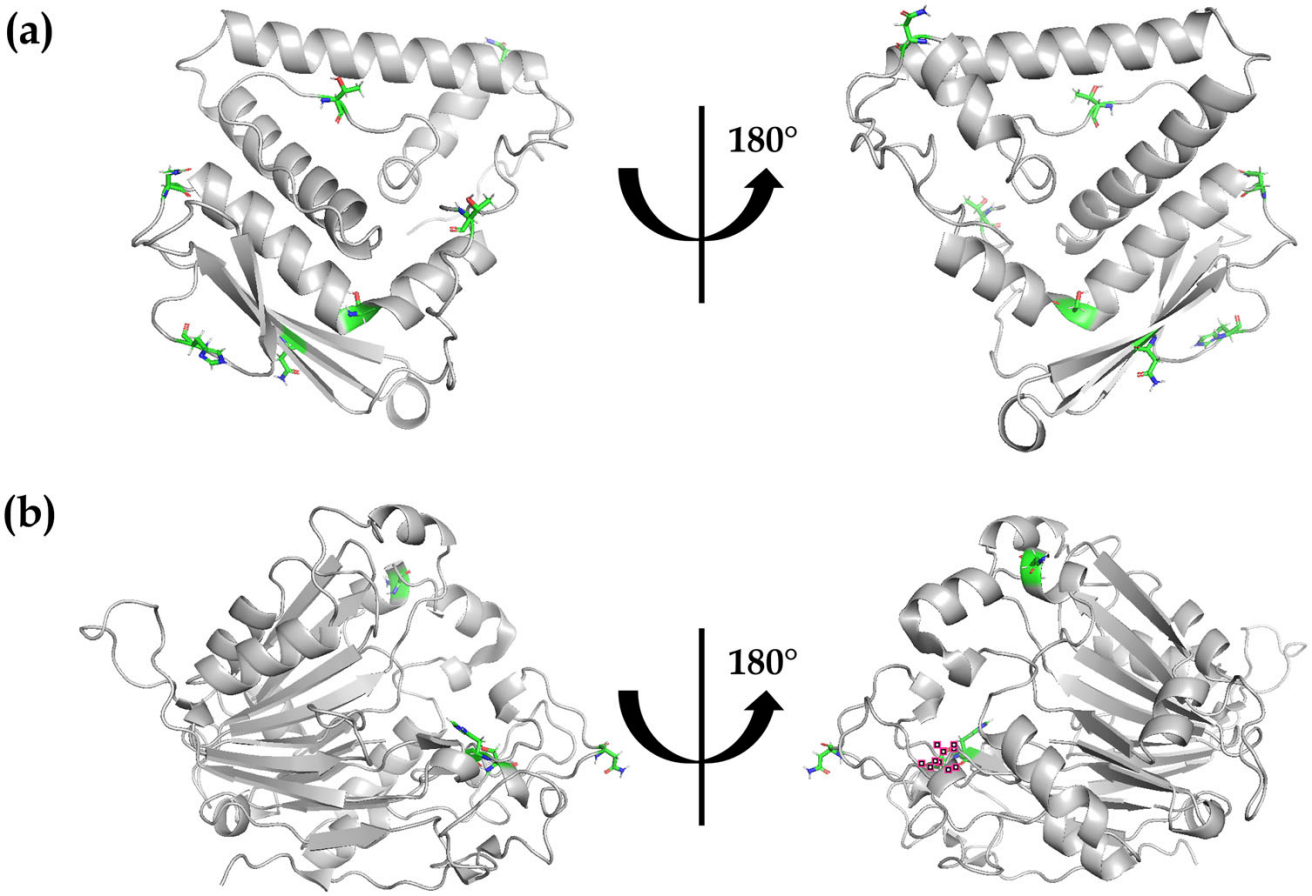
Structural analysis of the N‐terminal and C‐terminal peptides of CHO PLBL2 using a homology model predicted by AlphaFold2 using UniProt entry G3I6T1 (with the signaling sequences removed). Shown in color are residues which exhibit post‐translational modifications (including N‐linked glycosylation, O‐linked glycosylation, and phosphorylation). (a) Front and back views of the N‐terminal peptide. (b) Front and back views of the C‐terminal peptide.

As seen in Figure 7, structures of truncated CHO PLBL2 were also generated by removing residues L1 through C203 (rendering the C‐terminal peptide) and by removing residues S204 through D548 (rendering the N‐terminal peptide). Conformational changes due to truncation were interrogated using the energy minimization feature of the Molecular Operating Environment (version 2020. 0901). No significant conformational changes were observed for either the N‐terminal or C‐terminal peptide. Therefore, Figure 7 shows only the energy‐minimized structures of the N‐terminal and C‐terminal peptides, respectively.

Notably, in the last few years, the United States Pharmacopeial Convention (USP) released a form of recombinant CHO PLBL2 as an analytical reference material (ARM) to support assay development and quantitation of CHO PLBL2 in bioprocessing samples. The certificate of analysis for the USP CHO PLBL2 reports cleavage at S202/C203 (in our sequence numbering, corresponding to S239/C240 in the USP sequence numbering), which disagrees with the cleavage sequence we identified via peptide mapping (C203/S204 in our sequence numbering, corresponding to C240/S241 in the USP sequence numbering). This discrepancy may be attributed to the differences in production (e.g., expression, purification, and storage) and highlights the importance of in‐process controls. Furthermore, the enormous complexity of biologics calls for caution when relying on a single ARM.

### 4.5 CHO PLBL2 Is Heavily Decorated via Glycosylation and Phosphorylation

Peptide mapping also revealed that CHO PLBL2 is heavily decorated via N‐linked glycosylation, O‐linked glycosylation, and phosphorylation, as seen in the extensive mass spectral evidence in Figure S.11 through Figure S.25 in the Supporting Information. As summarized in Table 1 and as highlighted in Figure 1, five distinct N‐glycosylation sites were observed: N47, N69, N190, N395, and N474. Each site exhibited a plethora of distinct glycoforms, and all five sites possessed >98% total occupancy. The most abundant glycoform at each site included G2 (for N47), Man3F2 (for N69), G0F (for N190), Man5 (for N395), and G2F (for N474). Three of these sites are located on the N‐terminal peptide, which is generated by cleavage at C203/S204, while the other two are located on the resulting C‐terminal peptide. As seen in Figure S.26 through Figure S.30, the CHO PLBL2 structure predicted by AlphaFold2 suggests that all five N‐glycosylation sites reside on the outer surface of the folded protein and are solvent —exposed.

O‐linked glycosylation was observed at a single site: T3 (Table 1 and Figure 1). Six distinct glycoforms were observed at this site, yielding a total site occupancy of > 94% (Table 1). The most abundant glycoform was HexNAc_1_Hex_1_. As for the N‐glycosylation sites, the CHO PLBL2 structure predicted by AlphaFold2 suggests that T3 resides on the outer surface of the folded protein and is solvent exposed (see Figure S.31).

Finally, phosphorylation was identified at three different amino acids (rendering phosphohistidine, phosphoserine, and phosphothreonine) across five distinct sites: H39, S85, T167, S468, and H487. As seen in Table 1, site occupancy for phosphorylation—unlike that for N‐linked and O‐linked glycosylation—varied considerably by site, from as low as 2% for S468 to as high as 52% for S85. Similarly to the N‐glycosylation findings, the phosphorylation sites are distributed such that three sites are located on the N‐terminal peptide generated by cleavage at C203/S204, while the other two sites are on the C‐terminal peptide. As seen in Figure S.32 through Figure S.34, H39, H487, and T167 all reside on the outer surface of the folded protein and are solvent exposed. The structures predicted by AlphaFold2 suggest that S85 is buried within the core of intact CHO PLBL2 (as seen in Figure S.35) but becomes solvent exposed upon cleavage to yield the N‐terminal fragment (as seen in Figure S.36). In contrast, S468 is buried within the core of both intact CHO PLBL2 and the C‐terminal fragment (as seen in Figure S.37 and Figure S.38). Recent literature suggests that transient exposure of such sites because of protein dynamics may enable access and phosphorylation by kinases (Henriques and Lindorff‐Larsen 2020; Orioli et al. 2022). The low site occupancy (2%) of S468 suggests low solvent accessibility even when considering protein dynamics, while a 52% site occupancy for S85 suggests greater solvent accessibility even in the intact state, which may enable greater access by kinases.

As with the findings for the cleavage of CHO PLBL2, the certificate of analysis for the USP CHO PLBL2 indicates two potential N‐glycosylation sites (N227 and N511 in the USP sequence numbering, corresponding to N190 and N474 in our sequence numbering), while the other three sites we identified (N47, N69, and N395 in our sequence numbering, corresponding to N84, N106, and N432 in the USP sequence numbering) have not been reported. Moreover, the O‐glycosylation site (T3 in our sequence numbering, corresponding to T40 in the USP sequence numbering) and phosphorylation sites (H39, S85, T167, S468, and H487 in our sequence numbering, corresponding to H76, S122, T204, S505, and H524 in the USP sequence numbering) identified in the present work were not reported by USP.

## 5 Conclusion

Before this study, examinations of HCPs in biotherapeutics development had been limited primarily to identifying and cataloguing challenging species which copurify with biotherapeutics. Occasional studies have characterized enzyme activity for select species suspected of degrading polysorbates or other additives in protein formulations, but deeper interrogation of the fundamental nature of HCPs (e.g., proteoforms or variants) has essentially been overlooked, largely due to the lack of technical feasibility (i.e., ultra‐low abundance of HCPs of interest).

Enabled by our new enrichment method, the results of our battery of analyses showcase CHO PLBL2's exceptional heterogeneity and variant diversity and, moreover, the power of combining affinity enrichment with mass spectrometry and other methods to achieve deep structural characterization of intriguing bioprocessing impurities.

We see immediate implications for the analysis of CHO PLBL2 in protein‐based therapeutics. As discussed above, in the last few years, USP released a form of recombinant CHO PLBL2 as an ARM to support assay development and quantitation of CHO PLBL2 in bioprocessing samples. While the certificate of analysis for this ARM includes potential N‐glycosylation sites and a cleavage site, our detailed characterization work reveals not only additional N‐glycosylation sites but also previously unreported O‐glycosylation and phosphorylation sites. Moreover, our peptide mapping work enabled the confident assignment of CHO PLBL2 cleavage at C203/S204, which differs from that reported for the USP CHO PLBL2 at S202/C203 (both in our sequence numbering). We hope that our findings afford a more comprehensive and accurate panel of PTMs for the CHO PLBL2 ARM and analyses that depend on it.

We also see potential implications for downstream processing. In exhibiting a diverse array of amino acid sequences (e.g., variants produced via cleavage) and molecular decoration (e.g., glycoforms and phosphorylation, including partial occupancy of such PTMs), these CHO PLBL2 variants should necessarily exhibit different physicochemical properties and, potentially, disparate biological activities. We postulate that these physicochemical property differences may contribute to interactions between CHO PLBL2 and any co‐expressed biotherapeutics and, therefore, contribute to challenges separating CHO PLBL2 (or certain variants) during purification. To utilize this knowledge to aid downstream process development, we envision a framework analogous to that developed by Ranjan et al to address cathepsin D copurification with an IgG, in which detailed knowledge of the interacting surfaces of cathepsin D and the IgG led to a tailored and optimized wash buffer (an increase to pH 9) in the affinity capture step and, consequently, higher clearance of cathepsin D (Ranjan et al. 2019). Similarly, we postulate that more detailed structural and biochemical information about HCPs and their variants will give insight into differences in their biophysical properties and, necessarily, interaction with therapeutic proteins. Inspired by Ranjan et al. this structural knowledge could be combined with modeling and molecular dynamics simulations to characterize the interactions and devise targeted conditions (e.g., wash buffer pH changes or additives) to promote improved clearance of target impurities in existing unit operations. In extreme cases, an additional, targeted affinity capture step for an especially problematic HCP could be warranted. However, we only envision such a solution as a last‐resort effort where other potential solutions have been exhausted and such custom affinity resins become more commercially available or off the shelf.

Finally, while our work centered on PLBL2 derived from CHO, our affinity enrichment approach should be broadly applicable to enable the examination of countless other challenging HCPs from modalities and expression systems across the increasingly diverse bioprocessing landscape.

## Author Contributions

Michael E. Dolan and Lei (Leo) Wang contributed to the conceptualization and methodology, carried out experiments, interpreted the results, led the literature review, and prepared the manuscript. Alexander Tedeschi carried out experiments, interpreted the results, and prepared the manuscript. Yan Wang and Christopher Barton interpreted the results and reviewed and edited the manuscript. Sheldon F. Oppenheim contributed to the conceptualization and methodology, interpreted the results, and reviewed and edited the manuscript. Zhaohui Sunny Zhou contributed to the conceptualization and methodology, interpreted the results, and reviewed and edited the manuscript. All authors agreed to the final version of the manuscript.

## Conflicts of Interest

M.E.D., L.W., A.T., Y.W., C.B., and S.F.O. are employees of Takeda Development Center Americas, which is a wholly owned subsidiary of Takeda Pharmaceutical Company Ltd. All individuals may own Takeda stock, restricted stock units, and/or stock options.

## Supporting information

Supporting Info ‐ 25‐427.

## Data Availability

All data are incorporated into the article and its online supporting material.
