# Supplementary material for: Elucidation of Proteoforms of Chinese Hamster Ovary (CHO) Phospholipase B‐Like 2 (PLBL2) Captured From a Monoclonal Antibody
Source: Biotechnol Bioeng. 2025 Nov 10;123(2):406–19. doi: 10.1002/bit.70104 (PMC12779225; doi:10.1002/bit.70104)
Supplement: Supplementary file 1 — Supporting Info ‐ 25‐427. [file BIT-123-406-s001.pdf]

Supporting Information

Elucidation of Proteoforms of Chinese Hamster Ovary (CHO) Phospholipase B-Like 2 (PLBL2) Captured from a Monoclonal Antibody

Michael E. Dolan[1,2,3,†,*], Lei (Leo) Wang[4,†], Alexander Tedeschi[3], Yan Wang[4], Christopher Barton[4], Sheldon F. Oppenheim[3,*], Zhaohui Sunny Zhou[1,2,*]

AUTHOR ADDRESSES AND AFFILIATIONS:

[1] Department of Chemistry and Chemical Biology, Northeastern University, Boston, Massachusetts 02115, USA

[2] Barnett Institute for Chemical and Biological Analysis, Northeastern University, Boston, Massachusetts 02115, USA

[3] Biotherapeutics Process Development, Takeda Development Center Americas, 200 Shire Way, Lexington, Massachusetts, 02421 USA

[4] Analytical Development, Takeda Development Center Americas, 200 Shire Way, Lexington, Massachusetts 02421 USA

[†] These authors contributed equally to this work.

*Correspondence should be sent to: Michael E. Dolan (michael.dolan@takeda.com), Sheldon F. Oppenheim (sheldon.oppenheim@takeda.com), and Prof. Zhaohui Sunny Zhou (z.zhou@northeastern.edu)

Site-Specific Modification and Immobilization of Goat Anti-CHO PLBL2 Polyclonal IgG

Immobilization to Streptavidin Magnetic Beads

To prepare for immobilization, high-capacity streptavidin magnetic beads were buffer exchanged into 1X TBS (pH 7.4) according to the following: 5 mL of beads were resuspended (10 mg of beads per mL of solution) and transferred in equal-volume aliquots to four clean 1.5 mL Eppendorf tubes, which were placed onto the DynaMag™-2 magnet to magnetize and separate the beads from solution. The supernatant from each tube was discarded and replaced with 1,000 μL of 1X TBS (pH 7.4), into which the beads were resuspended. The tubes of beads were placed back onto the DynaMag™-2 magnet to magnetize and separate the beads from solution. The supernatants were discarded, and the process was repeated four more times. After discarding the final supernatants, the beads in each tube were resuspended into 250 μL of 1X TBS (pH 7.4) and combined into a single tube. The tube was placed back onto the DynaMag™-2 magnet to magnetize and separate the beads from solution, and the supernatant was discarded. The beads were then resuspended a final time in 267 μL of 1X TBS (pH 7.4) to achieve a concentration of ~150 mg of beads per mL of solution.

Assuming a ratio of 12 nmol of biotin per mg of streptavidin bead (as indicated by the vendor), about 1.1 mg of biotinylated anti-CHO PLBL2 IgG were mixed with the streptavidin beads at a molar ratio of 50:1 (streptavidin:biotin) and IgG concentration of 1.0 mg/mL (or 6.7 μM). The coupling contents were incubated using a MultiTherm heating/cooling shaker for 25 hours at 25°C while agitating at 1,000 rpm and shielding from light. Following immobilization, the beads were washed to remove any potentially uncoupled goat anti-CHO PLBL2 IgG. Washes were performed using 3 bead volumes (BVs; i.e., 3 x 300 μL) of 50 mM Tris (pH 8.0), 3 BVs of 50 mM Tris with 2 M sodium chloride (pH 8.0), and 3 BVs of 50 mM Tris (pH 8.0), as described

above. After washing, the beads were resuspended in 1.1 mL of 1X TBS (pH 7.4) to achieve an effective immobilized IgG concentration of 1.0 mg/mL (or 6.7 μM).

CHO HCP ELISA

The CHO PLBL2 content of the pembrolizumab samples before and after affinity capture was determined via CHO PLBL2 ELISA.

The ELISA kit was removed from storage at 2-8°C and equilibrated at room temperature for 1 hour prior to use. The pembrolizumab samples and PLBL2 assay control were removed from storage at ≤-65°C and thawed at room temperature.

The assay control was diluted 100X using diluent solution for a final working concentration of 3 ng/mL. The load sample of pembrolizumab was diluted 500X, then further serially diluted 1000X, 2000X, and 4000X using the diluent solution. The polished pembrolizumab sample was diluted 20X, then further serially diluted 40X, 80X, and 160X using the diluent solution.

Both the 100X detection antibody and 100X HRP-streptavidin solutions were diluted to working concentrations of 1X by adding 120 μL of the respective 100X solutions to 11.88 mL of diluent solution. The 1X wash solution was prepared by adding 50 mL of 20X wash solution to 950 mL of Milli-Q water.

The calibrator used to prepare the working standard curve was reconstituted using Milli-Q water, then diluted to a concentration of 20 ng/mL CHO PLBL2 in diluent solution according to the lot-specific certificate of analysis provided in the kit. Intermediate standard stock was prepared using a 2X dilution of the 20 ng/mL CHO PLBL2 calibrator in diluent solution for a final

concentration of 10 ng/mL. The working standard series was prepared by further diluting the 10 ng/mL intermediate standard stock in diluent solution to final standard concentrations of 5, 2.5, 1.25, 0.63, 0.31, 0.16, and 0.08 ng/mL.

100 µL of prepared standards, diluent blank, assay control, and pembrolizumab were transferred in triplicate to the microtiter strips in the ELISA plate, covered, and incubated on a plate shaker at 25°C for 2 hours while agitating at 500 rpm. Following the incubation, each well of the ELISA plate was washed 3 times with 375 µL of 1X wash solution using a Bio-Tek automated plate washer (ELX405TS from Agilent; Santa Clara, CA, USA).

100 µL of 1X detection antibody were added to each well of the microtiter strips in the ELISA plate. The plate was covered with a foil plate seal and incubated on a plate shaker at 25°C for 20 minutes while agitating at 500 rpm. Following the incubation, each well of the ELISA plate was washed using the same wash procedure described above.

100 µL of 1X HRP-streptavidin were added to each well of the microtiter strips in the ELISA plate. The plate was covered with a foil plate seal and incubated on a plate shaker at 25°C for 20 minutes while agitating at 500 rpm. Following the incubation, each well of the ELISA plate was washed using the same wash procedure described above.

100 µL of TMB chromogen substrate solution were added to each well of the microtiter strips in the ELISA plate. The plate was covered with a foil plate seal and incubated on a plate shaker at 25°C for 10 minutes while agitating at 500 rpm. Following the incubation, the reaction was stopped by adding 100 µL of stop solution to each well of the ELISA plate. The absorbance at 450 nm of each well was measured using a SpectraMax M2E microplate reader (Molecular Devices; San Jose, CA, USA).

The data were analyzed using SoftMax Pro (Molecular Devices, version 7.0.3). The standard curve was generated by plotting the absorbance at 450 nm against the CHO PLBL2 concentration of the standards and applying a 4-parameter logistic fit. Each sample and assay control replicate were interpolated against this fitted standard curve and multiplied by the initial dilution factor to determine the CHO PLBL2 concentration. All replicates that fell within the standard curve range of 5 to 0.08 ng/mL were used to determine the final back-calculated CHO PLBL2 concentration of the samples and assay control. A minimum of two dilutional levels were on curve and included in the analysis for each of the pembrolizumab samples.

Analysis of Molecular Size Isoforms via Capillary Western Blot

The CHO PLBL2 content of the pembrolizumab starting material (i.e., load), pembrolizumab after affinity capture (i.e., depleted of CHO PLBL2), and strip (or enriched) fractions (i.e., enriched and recovered CHO PLBL2) was analyzed via capillary Western blot using a procedure similar to that previously described (Dolan et al., 2024). The analysis was performed using the Jess (Protein Simple, 004-650) using a 12 – 230 kDa separation module and the anti-mouse detection module. Samples of the load, depleted, and strip fractions were prepared either undiluted or diluted. The standard curve of recombinant CHO-S PLBL2 (enabling quantitation of CHO PLBL2 in the pembrolizumab samples) was prepared with 100 ng/µL BSA functioning as a carrier protein; standards were administered at 50 pg, 25 pg, 10 pg, 5 pg, and 1 pg of CHO-S PLBL2. Three negative controls were included: first, a control capillary in which the 25 pg CHO-S PLBL2 standard served as the antigen but the primary mouse anti-CHO PLBL2 monoclonal antibody was replaced with antibody diluent; second, a control capillary in which

the 25 pg CHO-S PLBL2 standard served as the antigen but the anti-mouse HRP conjugate was replaced with antibody diluent; and, finally, a control capillary in which the antigen was replaced by 0.1X sample buffer while the primary mouse anti-CHO PLBL2 monoclonal antibody and anti-mouse HRP conjugate were unchanged.

Separation was performed using default settings, as described previously (Dolan et al., 2024). The separation and immunoblotting were analyzed using Compass for Simple Western (version 4.0.0). Peaks were detected and identified automatically by the software, but each electropherogram was inspected and manual corrections to standards were performed, where necessary. For the CHO-S PLBL2 standards, peak areas of the dominant PLBL2 peak (~80 kDa) were determined, plotted, and fitted using linear regression. Using the standard curve, the limit of quantitation (LOQ) for the capillary Western blot was determined to be <1 pg CHO-S PLBL2. Areas of the analogous ~80 kDa peaks in the load, depleted, and strip fractions were then used to determine their CHO PLBL2 content.

Analysis of Charge Isoforms via Isoelectric Focusing (IEF) Gel and Western Blot

To enable IEF under native conditions, two Novex™ IEF gels (pH 3-7) were prepared. The three strip fractions from the affinity capture were each mixed with a 2X concentrate of IEF sample buffer (pH 3-7). Lanes assigned to these samples were loaded with 20 µL of prepared sample. In contrast, lanes assigned to IEF marker (pH 3-10) were loaded with 10 µL of IEF marker. Electrophoresis was conducted using an XCell SureLock® Mini-Cell connected to a Criterion™ PowerPac Universal power supply by running at 100 V for 1 hour, 200 V for 1 hour, and 500 V for 30 minutes.

The IEF gels were then removed and rinsed using deionized water. One IEF gel was incubated in 1X IEF fixing solution while agitating at room temperature (about 22°C) for 30 minutes. After fixing, the IEF gel was rinsed using water, and protein bands were stained by incubating the gel with One-Step Blue® protein gel stain at room temperature overnight, followed by destaining in water for 2 hours. Bands were visualized using a GS-900 Calibrated Densitometer from Bio-Rad. The imaging system used the Coomassie Brilliant Blue R-250 setting, employing a transmissive scanning mode and a red filter.

The second IEF gel was equilibrated in transfer buffer (0.7% (w/v) acetic acid, pH 3.0) for 10 minutes while preparing the Western blot. Transfer of the proteins from the IEF gel to the PVDF membrane was performed via wet transfer. Prior to use, the PVDF membrane was immersed in methanol for 2 minutes before dripping to remove excess methanol and being transferred to the transfer buffer for 5 minutes. Similarly, the filter papers and sponges were immersed in transfer buffer for 5 minutes. The transfer sandwich was assembled in the XCell II™ Blot Module with the PVDF membrane on the negatively charged electrode side of the stack and the IEF gel on the positively charged electrode side of the stack, according to vendor instructions. The XCell II™ Blot Module was then assembled in the XCell SureLock® Mini-Cell, with the upper chamber filled with transfer buffer and the lower chamber filled with deionized water. Using the Criterion™ PowerPac Universal power supply, protein transfer proceeded at 20 V for 1 hour.

Blocking was performed by incubating the PVDF membrane in phosphate-buffered saline containing 0.1% polysorbate 20 (i.e., PBST) with 5% dry milk while gently agitating at 2-8°C overnight.

After decanting the blocking solution, the primary antibody solution (0.1 µg/mL of the mouse anti-CHO PLBL2 monoclonal IgG in PBST) was applied and the PVDF membrane was

incubated while gently agitating at room temperature for 60 minutes. After incubation, the primary antibody solution was decanted and the PVDF membrane was washed three times using PBST while gently agitating at room temperature for 5 minutes per wash.

After the final wash, the secondary antibody solution (0.1 µg/mL of the donkey anti-mouse polyclonal IgG-HRP conjugate in PBST) was applied and the PVDF membrane was incubated while gently agitating at room temperature for 60 minutes. After incubation, the secondary antibody solution was decanted and the PVDF membrane was washed three times using PBST while gently agitating at room temperature for 30 seconds per wash, followed by three more washes in the same solution for 10 minutes per wash.

Immediately prior to use, the working ECL reagent was prepared by mixing ECL Reagent 1 and Reagent 2 in equal-volume amounts. After decanting the final PBST wash following secondary antibody incubation, the working ECL reagent was added to the PVDF membrane, and the membrane was incubated while gently agitating at room temperature for 2 minutes to ensure universal coverage of the membrane by the ECL reagent. The membrane was then removed using forceps and excess ECL reagent was allowed to drip off just prior to imaging.

Imaging employed a G:Box Chemi-XX6 imaging system equipped with Genesis 1.8.2.0 (Syngene). Protein bands were visualized using the ECL chemiluminescent blot setting and exposure times in an additive series which included 1 second, 2 seconds, 5 seconds, 10 seconds, 30 seconds, 1 minute, 2 minutes, and 5 minutes.

Peptide Mapping Analysis

Sample Preparation

Samples were prepared with two digestion replicates as follows: (1) Load: pembrolizumab starting material, (2) Depleted: pembrolizumab after custom affinity capture (i.e., depleted of endogenous CHO PLBL2), (3) Enriched: pool of enriched fractions (i.e., enriched and recovered endogenous CHO PLBL2), (4) USP: USP recombinant CHO PLBL2 (Catalog #1582716, Lot #F174K0), and (5) iCLlab: Immunology Consultants Laboratory recombinant CHO-S PLBL2 (Catalog #AG65-0365Z, Lot #5). The total protein concentration for each sample was measured in triplicate using NanoDrop A280 with the IgG workflow. A volume of each sample containing 50 µg of total protein was aliquoted and dried overnight at 5°C in a CentriVap vacuum concentrator (Labconco, Kansas City, MO, USA). Before digestion, each aliquot was redissolved in 20 µL of denaturing buffer (5% SDS, 100 mM Tris, pH 7.5). Two 50 µg aliquots were prepared per sample as digestion duplicates. Digestion followed the ProtiFi S-Trap protocol with minor modifications. Proteins were reduced with 5 mM DTT at 70°C for 45 minutes and alkylated with 10 mM IAM at 25°C for 30 minutes while shielding from light. After alkylation, the samples were acidified with 1.2% phosphoric acid, and proteins were precipitated using binding buffer (90% methanol, 100 mM TEAB). The precipitated proteins were loaded onto ProtiFi S-Trap micro spin columns (ProtiFi, Fairport, NY, USA) and subjected to three wash and centrifugation cycles with binding buffer. Digestion was performed using 20 µL of 0.2 µg/µL trypsin (reconstituted in 50 mM TEAB buffer) at an enzyme-to-protein ratio of 1:12.5, incubating overnight at 37°C. Peptides were sequentially eluted with 50 mM TEAB, 0.1% formic acid, and 50% acetonitrile/0.1% formic acid, followed by drying in a CentriVap vacuum concentrator and reconstitution in 100 µL of 0.1% (w/v) formic acid. The digests were centrifuged and transferred to glass vials for LC-MS/MS analysis, with each digest injected in duplicate as described in the Materials and Methods section.

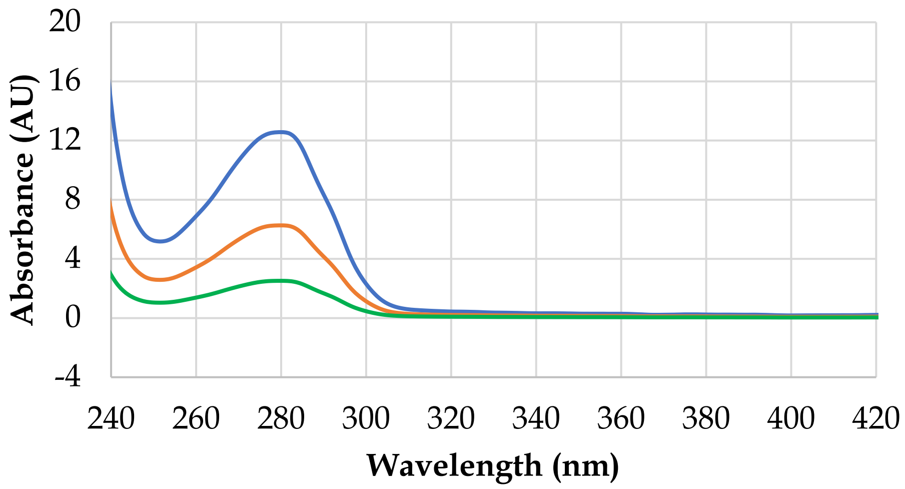

Figure S.1. Overlay of UV-Vis spectra of the native goat polyclonal anti-PLBL2 IgG at three different dilutions, showing an absorbance maximum at 280 nm. The extinction coefficient is 1.4 mL mg^{-1} cm^{-1} at 280 nm. All measurements utilized Lunatic plate frame strips with a linear range of 0.03 – 275 OD. Absorbances were normalized by the analysis software to a 1 cm path length.

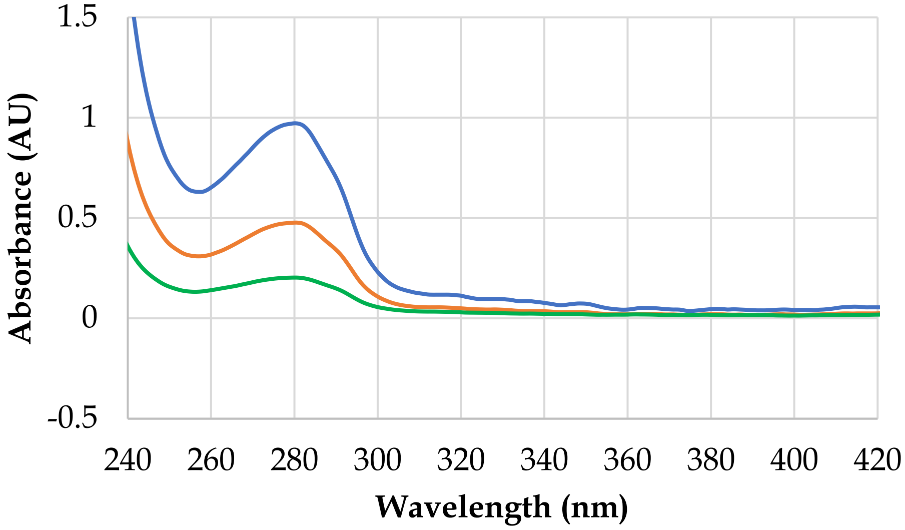

Figure S.2. Overlay of UV-Vis spectra of the phospholipase B-like 2 (PLBL2) from Chinese hamster ovary cells (of the CHO-S lineage) at three different dilutions, showing an absorbance maximum at 280 nm. The extinction coefficient is 2.0 mL mg^{-1} cm^{-1} at 280 nm. All measurements utilized Lunatic plate frame strips with a linear range of 0.03 – 275 OD. Absorbances were normalized by the analysis software to a 1 cm path length.

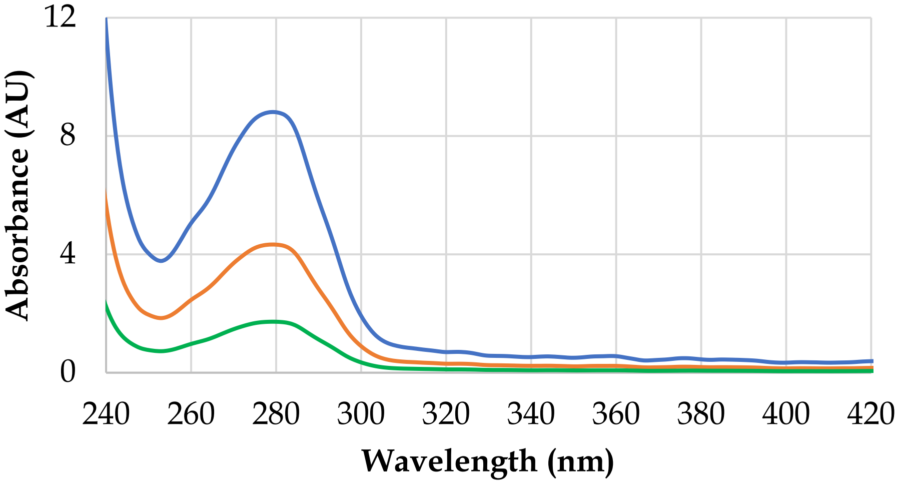

Figure S.3. Overlay of UV-Vis spectra of pembrolizumab (a humanized monoclonal IgG4 antibody, recombinantly expressed using Chinese hamster ovary cells) at three different dilutions, showing an absorbance maximum at 280 nm. The extinction coefficient is 1.41 mL mg^{-1} cm^{-1} at 280 nm. All measurements utilized Lunatic plate frame strips with a linear range of 0.03 – 275 OD. Absorbances were normalized by the analysis software to a 1 cm path length.

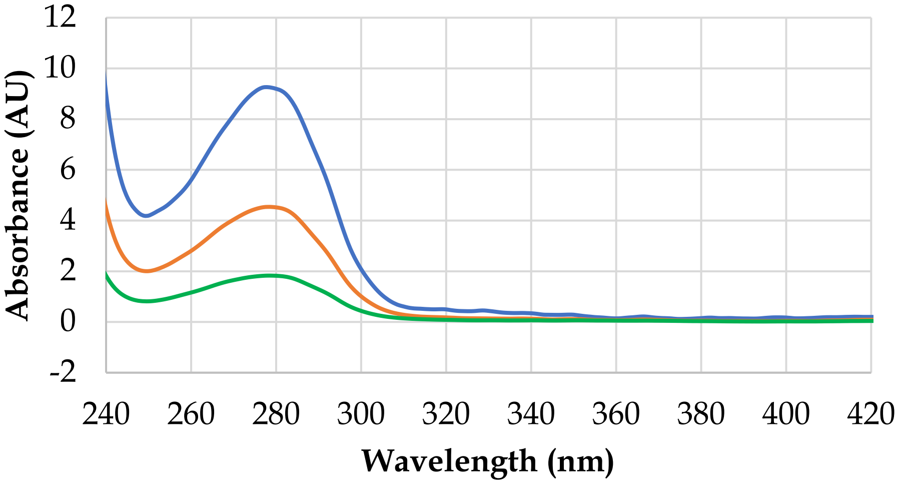

Figure S.4. Overlay of UV-Vis spectra of the native mouse monoclonal anti-PLBL2 IgG at three different dilutions, showing an absorbance maximum at 280 nm. The extinction coefficient is 1.4 mL mg^{-1} cm^{-1} at 280 nm. All measurements utilized Lunatic plate frame strips with a linear range of 0.03 – 275 OD. Absorbances were normalized by the analysis software to a 1 cm path length.

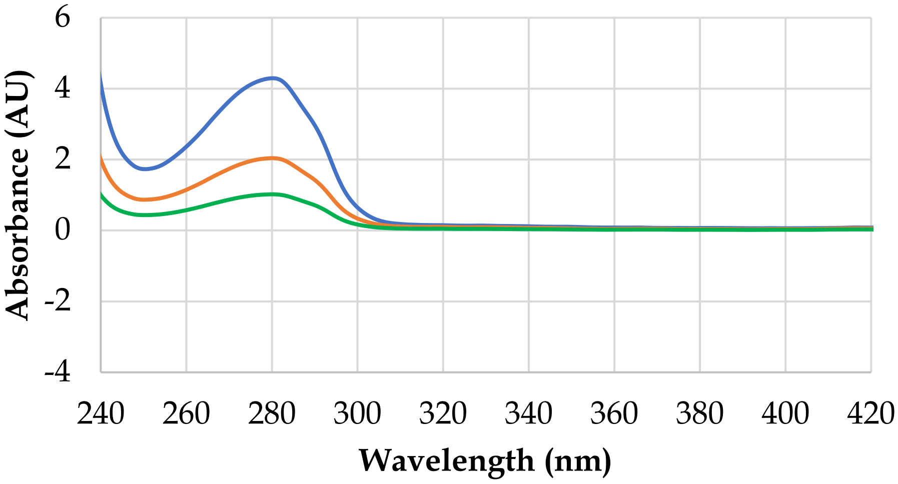

Figure S.5. Overlay of UV-Vis spectra of the United States Pharmacopoeia (USP) phospholipase B-like 2 (PLBL2) from Chinese hamster ovary cells at three different dilutions, showing an absorbance maximum at 280 nm. The extinction coefficient is 2.0 mL mg^{-1} cm^{-1} at 280 nm. All measurements utilized Lunatic plate frame strips with a linear range of 0.03 – 275 OD. Absorbances were normalized by the analysis software to a 1 cm path length.

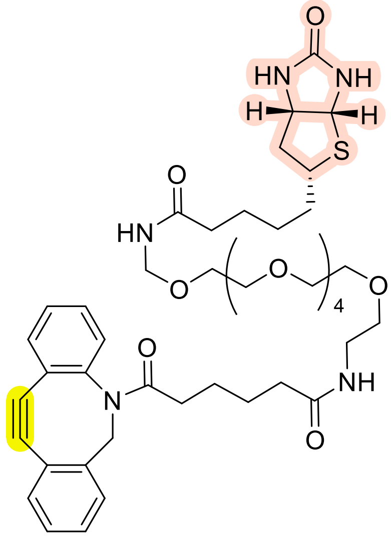

Figure S.6. Structure of biotin-PEG$_4$-DBCO, where the biotin moiety is shown in light pink at the top of the structure and the strained alkyne is shown in yellow on the left side of the dibenzocyclooctyne moiety.

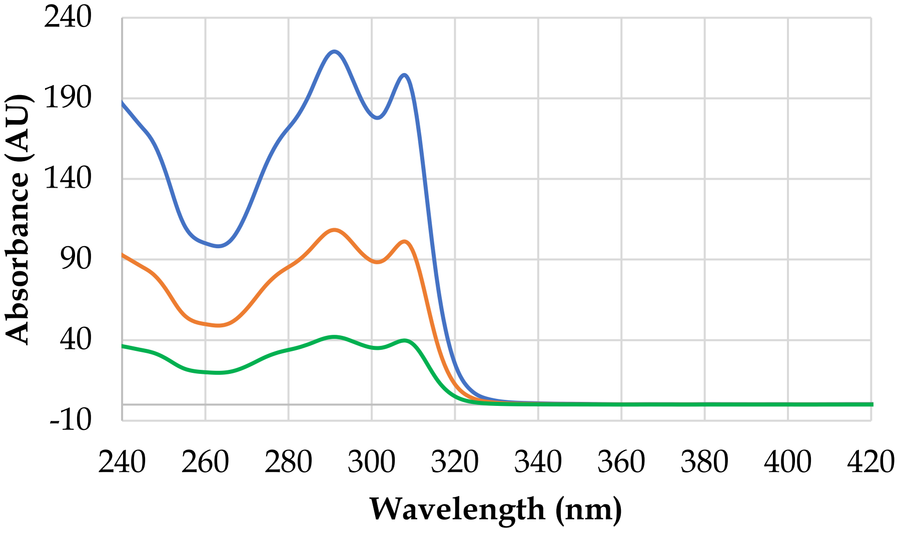

Figure S.7. Overlay of UV-Vis spectra of DBCO-PEG4-biotin at three different dilutions, showing a local absorbance maximum at 307 nm. The extinction coefficient is 20,000 M^{-1} cm^{-1} at 307 nm. All measurements utilized Lunatic plate frame strips with a linear range of 0.03 – 275 OD. Absorbances were normalized by the analysis software to a 1 cm path length.

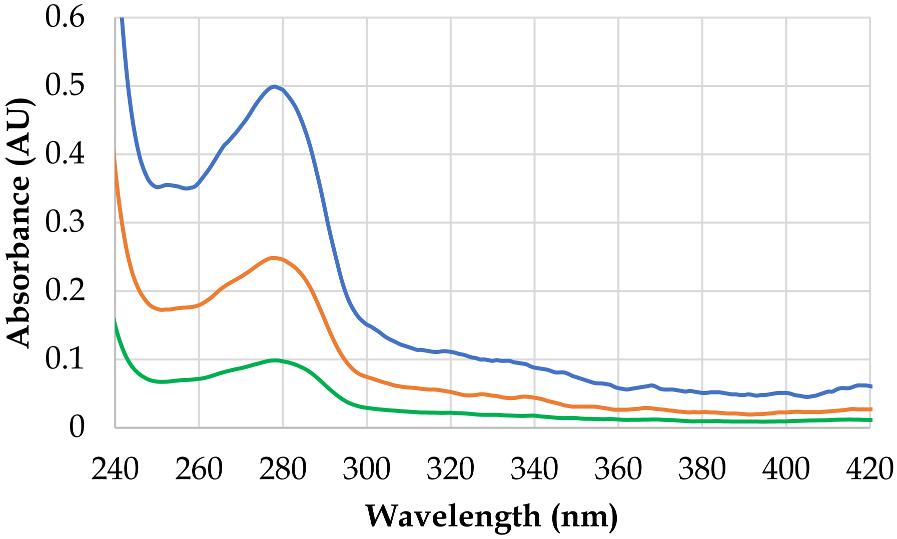

Figure S.8. Overlay of UV-Vis spectra of bovine serum albumin at three different dilutions, showing absorbance maxima at 280 nm. The extinction coefficient is 0.67 mL mg^{-1} cm^{-1} at 280 nm. All measurements utilized Lunatic plate frame strips with a linear range of 0.03 – 275 OD. Absorbances were normalized by the analysis software to a 1 cm path length.

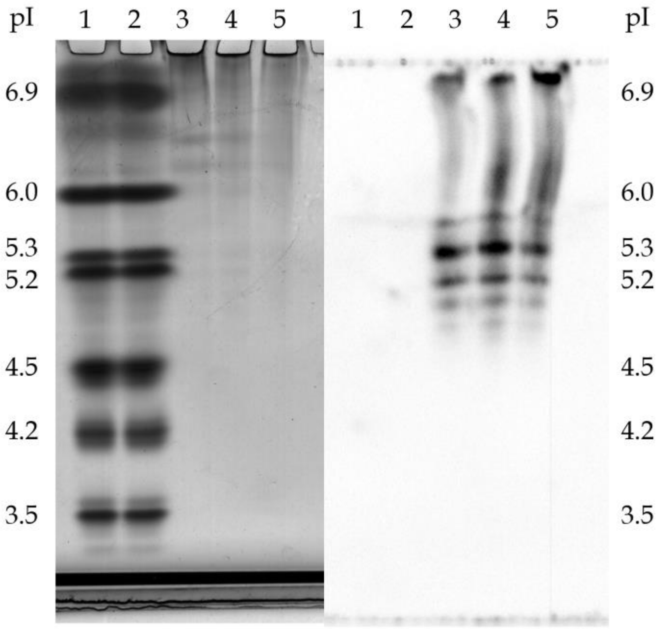

Figure S.9. Analysis via pH 3-7 isoelectric focusing (IEF) gel (left half) and Western blot (right half) of the charge variants of endogenous CHO PLBL2 captured from pembrolizumab. Lanes 1 and 2: isoelectric point (pI) markers. Lanes 3 through 5: CHO PLBL2 captured, enriched, recovered from pembrolizumab. Numerous distinct protein bands are observed between pIs of about 5.0 and about 6.0, indicating a diverse array of charge isoforms for endogenous CHO PLBL2 within this pI range. Bands are also observed at pIs >7.0, which were not fully resolved. For reference, the theoretical pI for CHO PLBL2 (calculated based on the amino acid sequence of UniProt entry G3I6T1) is 5.63.

(a)

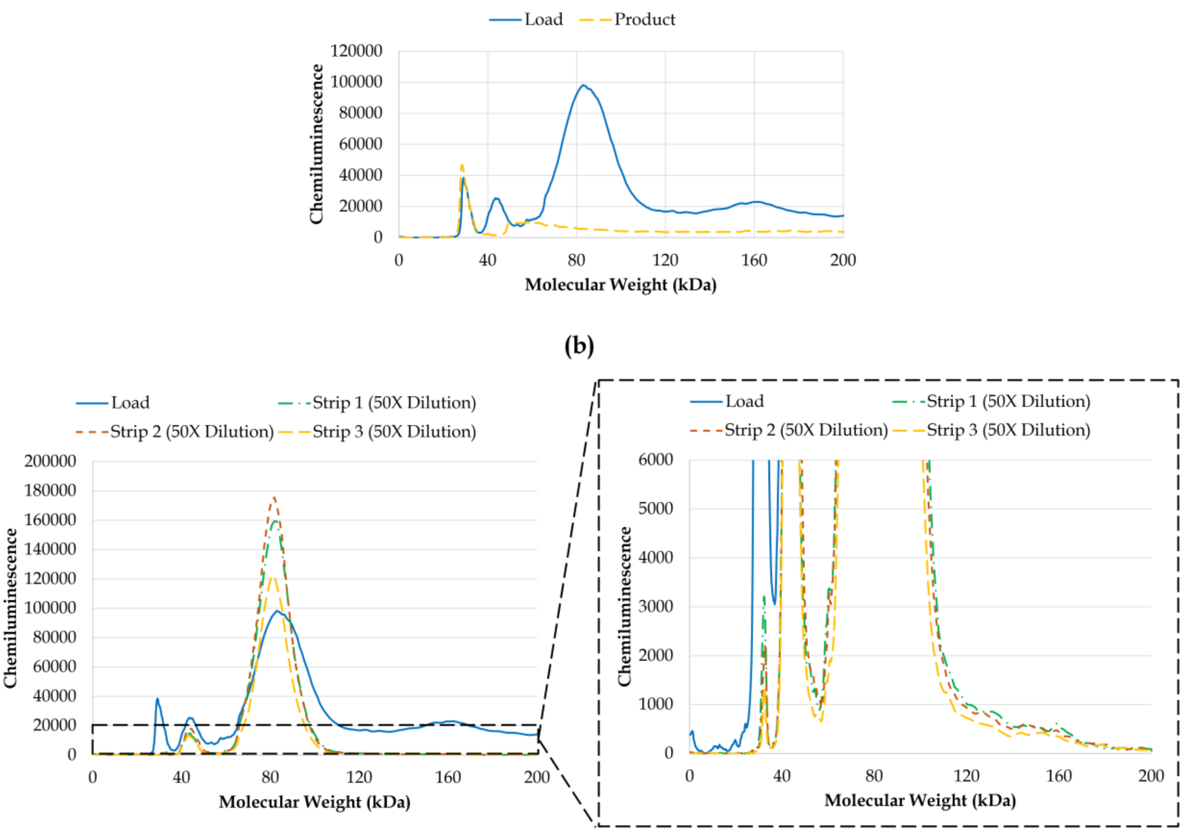

Figure S.10. Electropherograms from the capillary Western blot analysis of endogenous CHO PLBL2 capture from pembrolizumab. (a) Profiles for pembrolizumab pre-capture ("load") and post-capture ("product"). The electropherogram for the load exhibits major peaks at ~80 kDa, ~45 kDa, and ~30 kDa, confirming the presence of residual endogenous CHO PLBL2. The electropherogram for the product exhibits no peak at ~80 kDa, indicating successful capture of the CHO PLBL2 by the immobilized IgG. (b) Profiles for the strips in which CHO PLBL2 was recovered from the immobilized IgG by treatment using 1% (w/v) formic acid (pH 2.2). The electropherograms for the strips show the expected major and minor peaks at ~80 kDa and ~45 kDa, respectively, indicating successful recovery of the CHO PLBL2 previously bound to the resin.

N47 Glycopeptide: LVDGIHPYAVAWA**N**(G2, HexNAc4Hex5)LTN

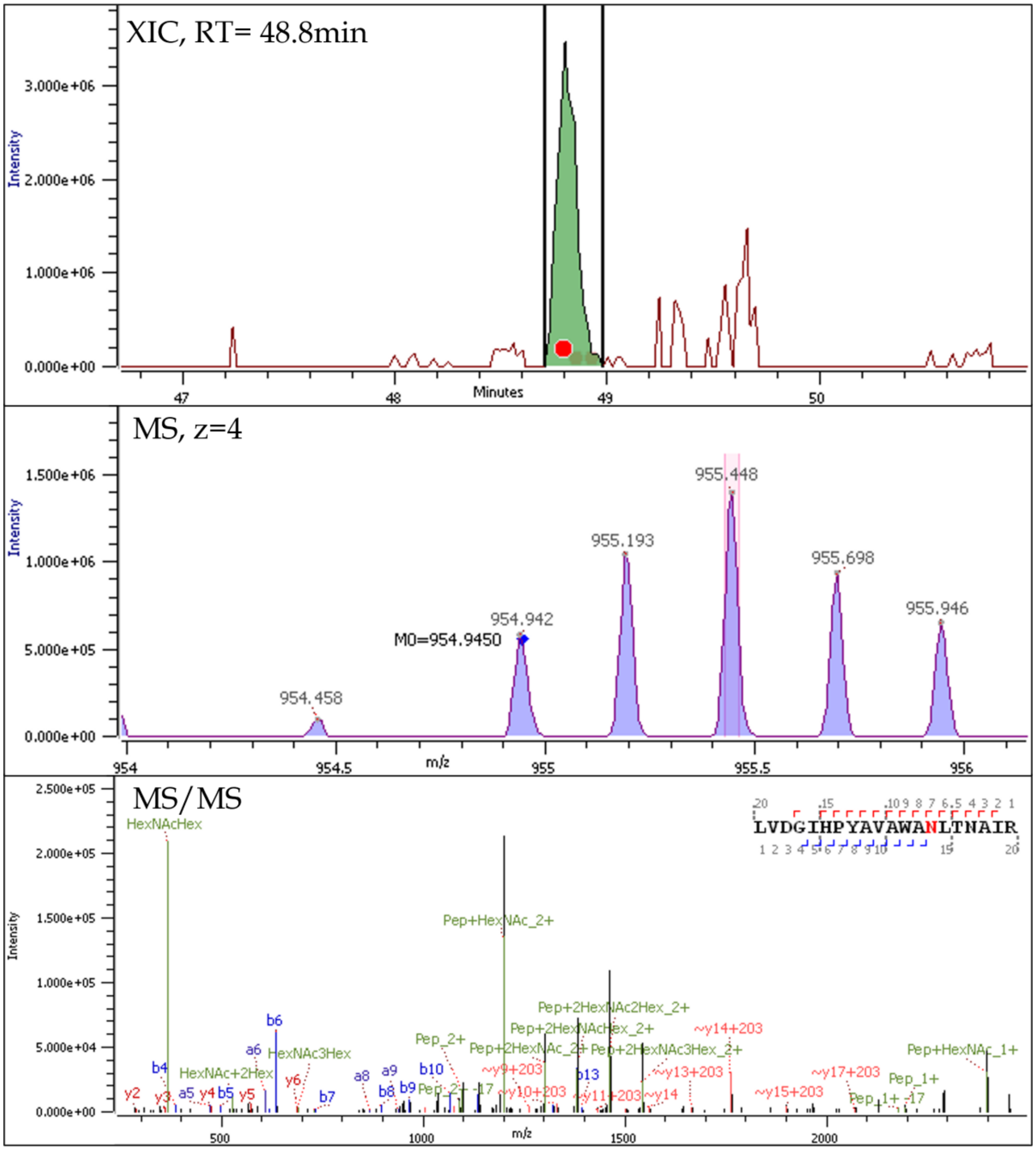

Figure S.11. Representative extracted ion chromatogram (XIC), mass spectrum (MS), and annotated tandem mass spectrum (MS/MS) for the identified peptide exhibiting glycosylation at residue N47.

N47 Aglycosylated Peptide: LVDGIHPYAVAWA**N**LTN

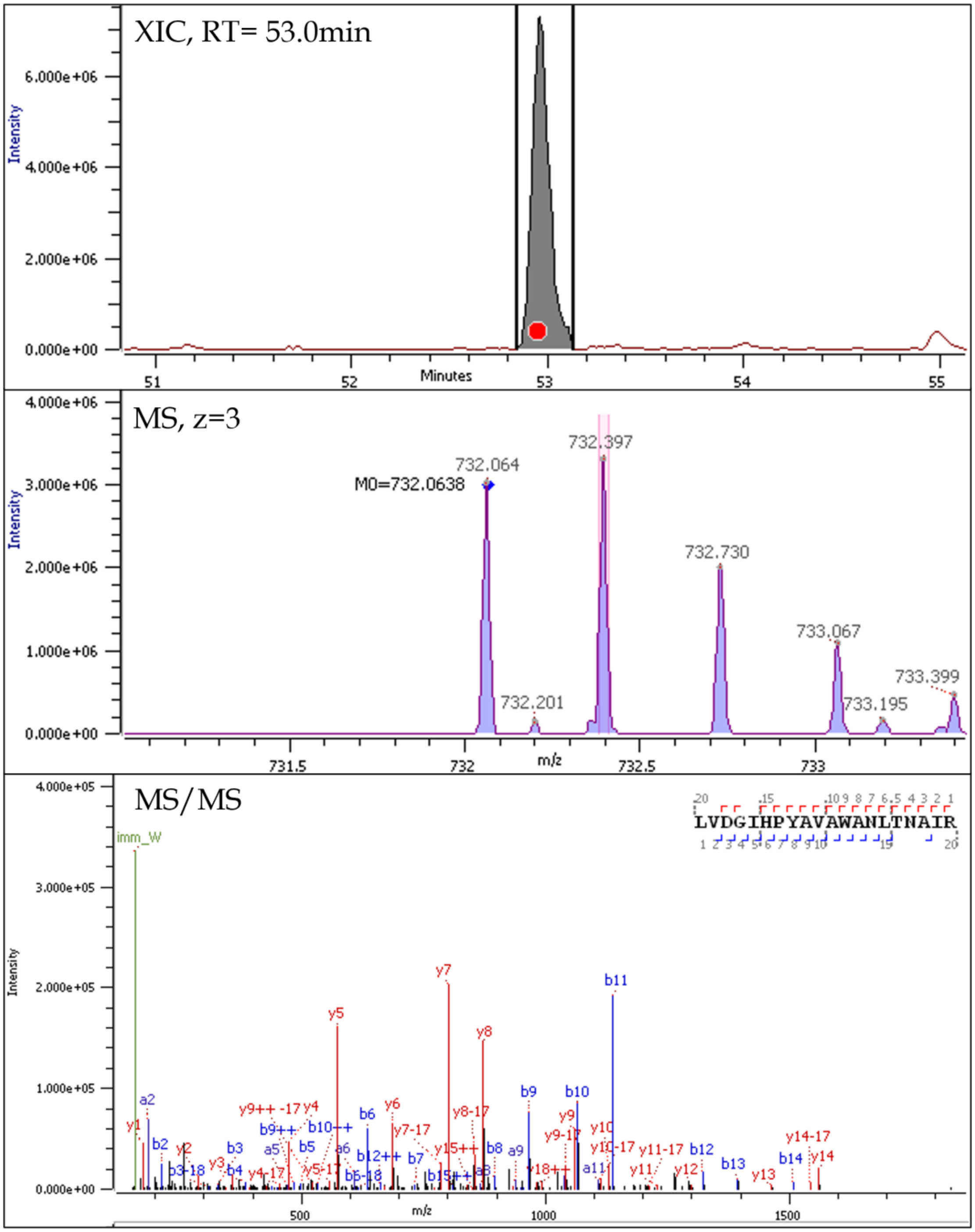

Figure S.12. Representative extracted ion chromatogram (XIC), mass spectrum (MS), and annotated tandem mass spectrum (MS/MS) for the identified peptide exhibiting no glycosylation at residue N47.

N69 Glycopeptide: ETGWAYLDLGTNGSYN(Man5, HexNAc2Hex5) DSLQAYAAGVVEASVSEELIYMHWMNTMVNYCGPFEYEVGYCEK

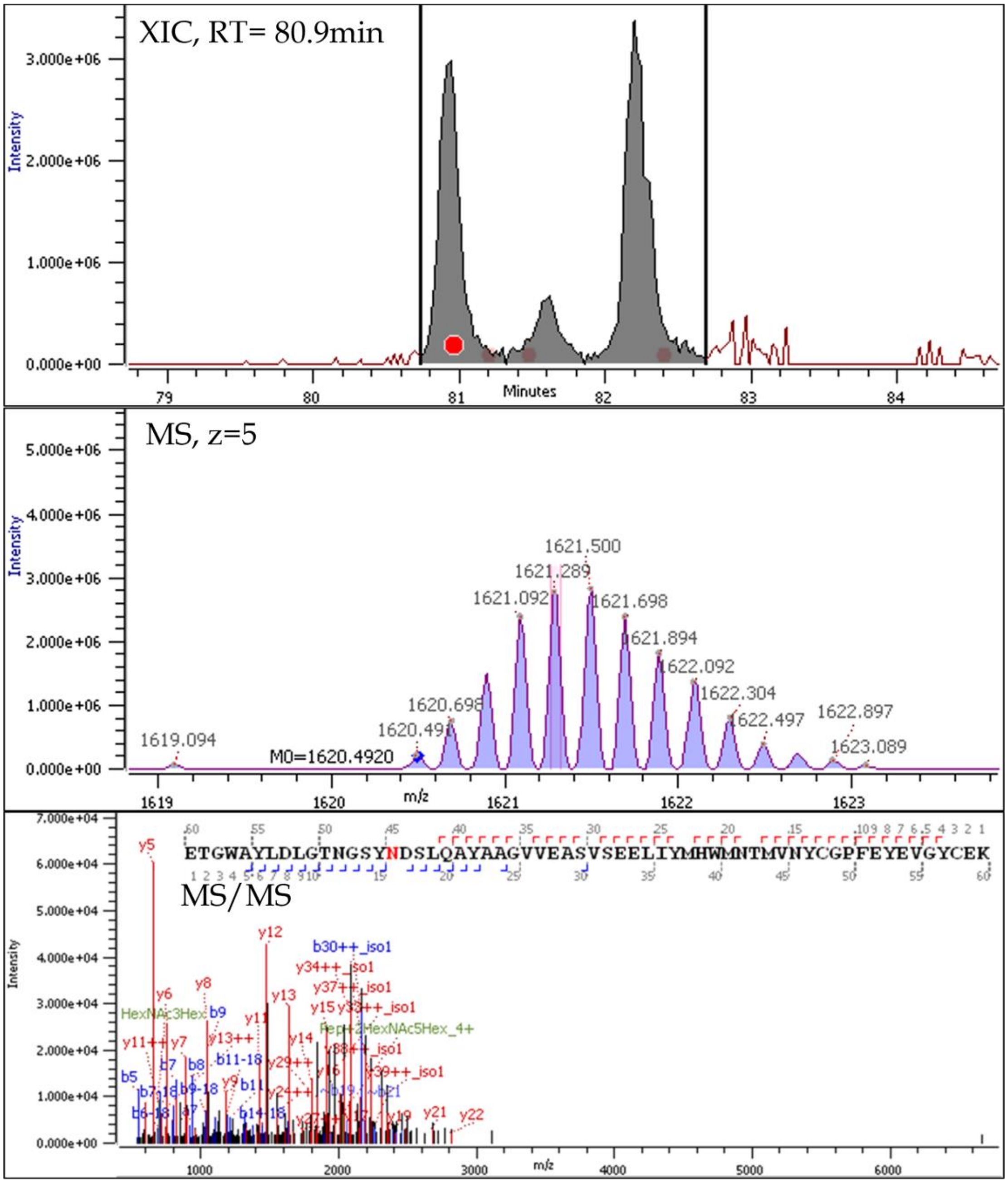

Figure S.13. Representative extracted ion chromatogram (XIC), mass spectrum (MS), and annotated tandem mass spectrum (MS/MS) for the identified peptide exhibiting glycosylation at residue N69.

N190 Glycopeptide: FTIKPLGFLLLQIAGDLEDLEQAL**N**(G0F, HexNAc4Fuc1Hex3)K

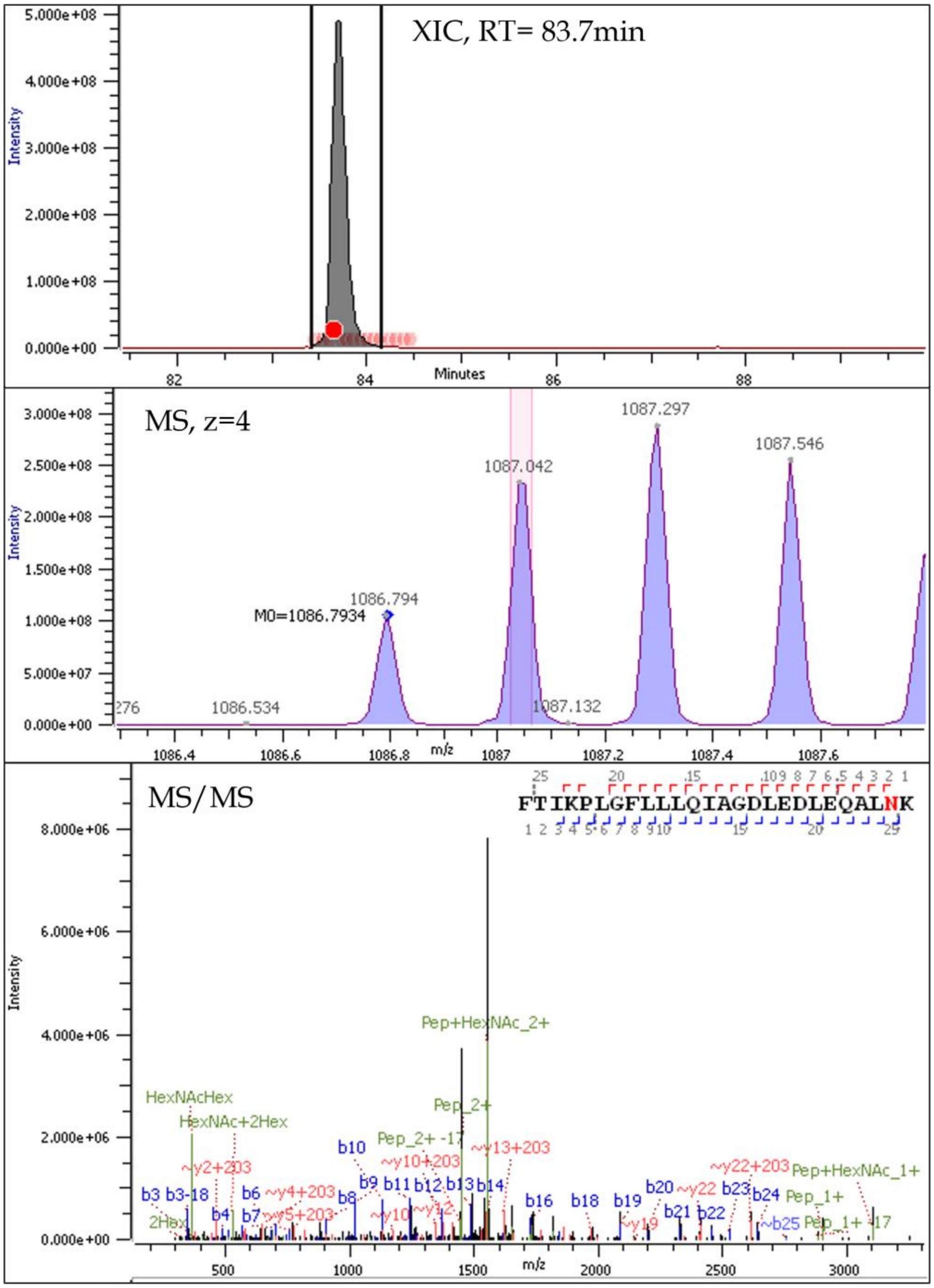

Figure S.14. Representative extracted ion chromatogram (XIC), mass spectrum (MS), and annotated tandem mass spectrum (MS/MS) for the identified peptide exhibiting glycosylation at residue N190.

N190 Aglycosylated Peptide: FTIKPLGFLLLQIAGDLEDLEQALNK

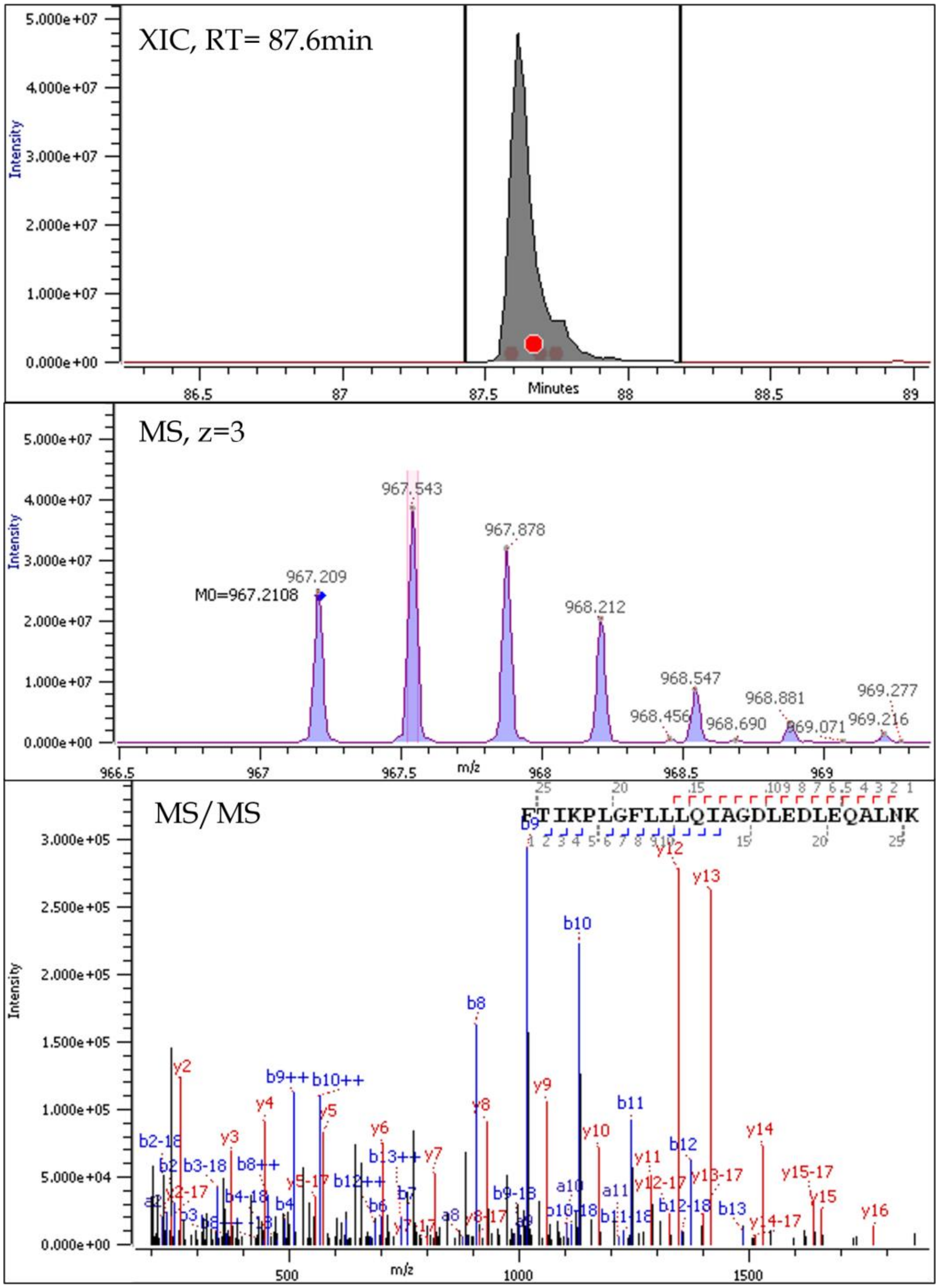

Figure S.15. Representative extracted ion chromatogram (XIC), mass spectrum (MS), and annotated tandem mass spectrum (MS/MS) for the identified peptide exhibiting no glycosylation at residue N190.

N395 Glycopeptide:
TTYWASYNIPFFEIVF**N**(Man5, HexNAc2Hex5)ASGLQDLVAQYGDWFSYTK

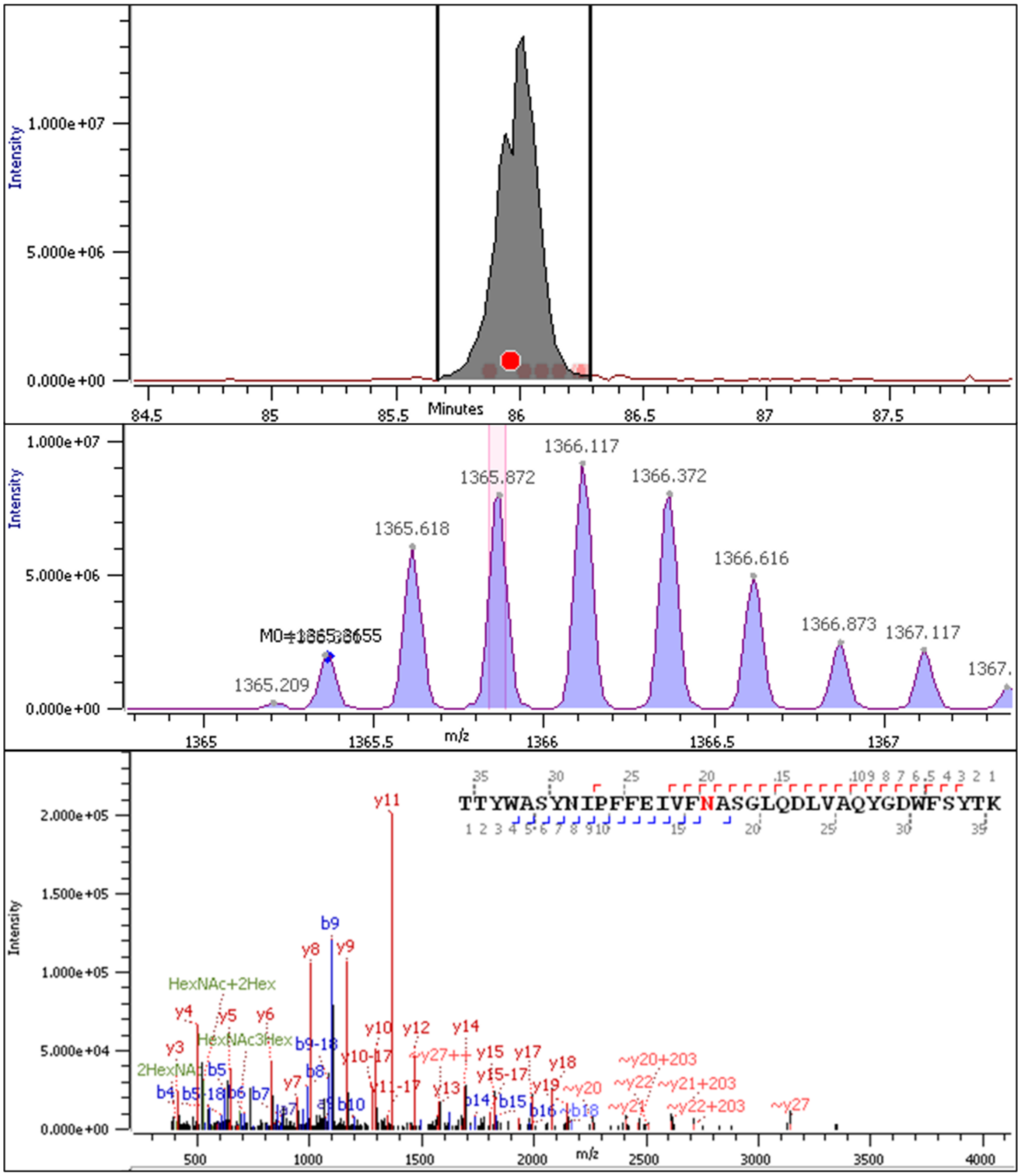

Figure S.16. Representative extracted ion chromatogram (XIC), mass spectrum (MS), and annotated tandem mass spectrum (MS/MS) for the identified peptide exhibiting glycosylation at residue N395.

N395 Aglycosylated Peptide:
TTYWASYNIPFFEIVFNASGLQDLVAQYGDWFSYTK

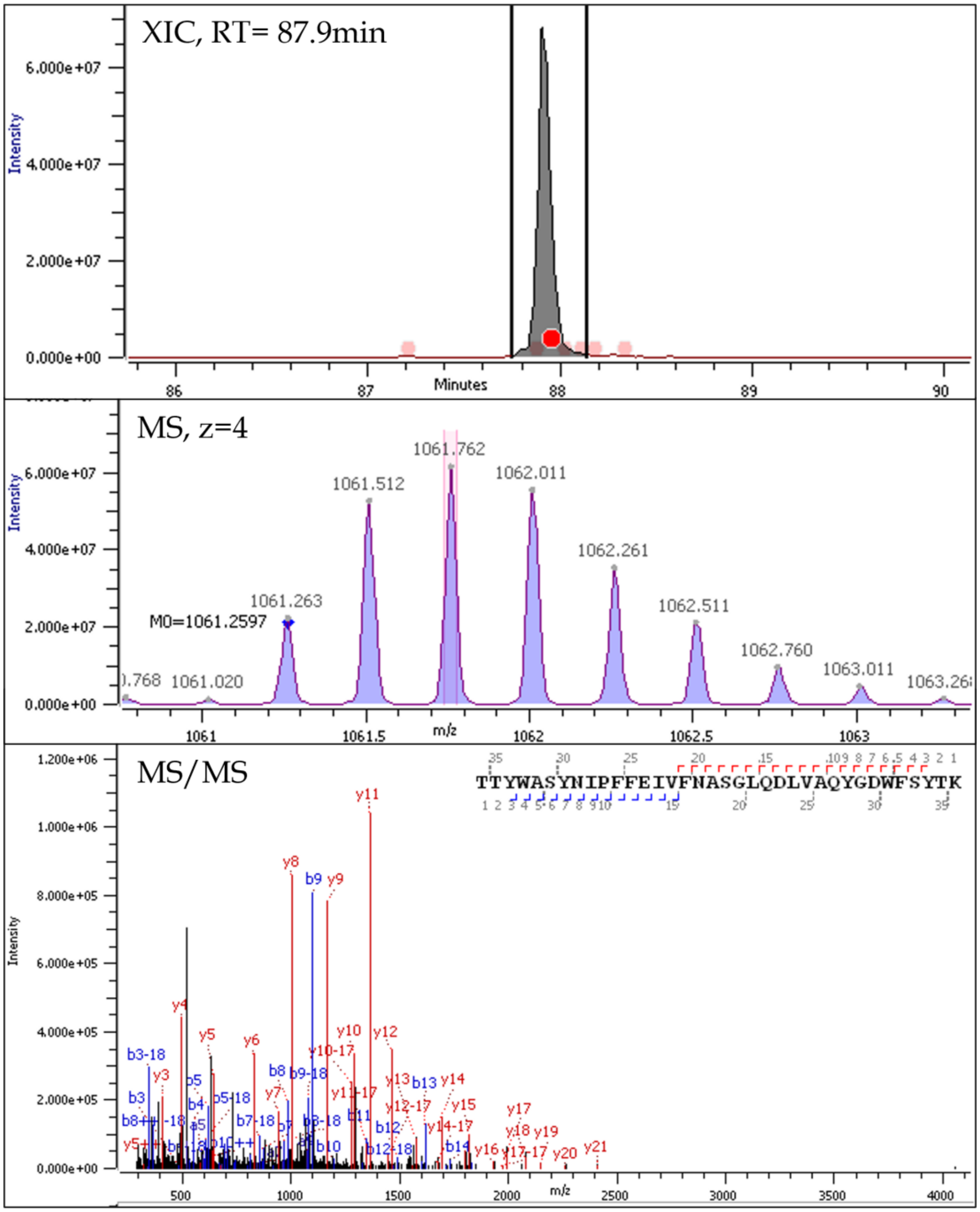

Figure S.17. Representative extracted ion chromatogram (XIC), mass spectrum (MS), and annotated tandem mass spectrum (MS/MS) for the identified peptide exhibiting no glycosylation at residue N395.

N474 Glycopeptide: SDLNPA**N**$_{(G2F,\ HexNAc4Fuc1Hex5)}$ GSYPFQALYQRPHGGIDVK

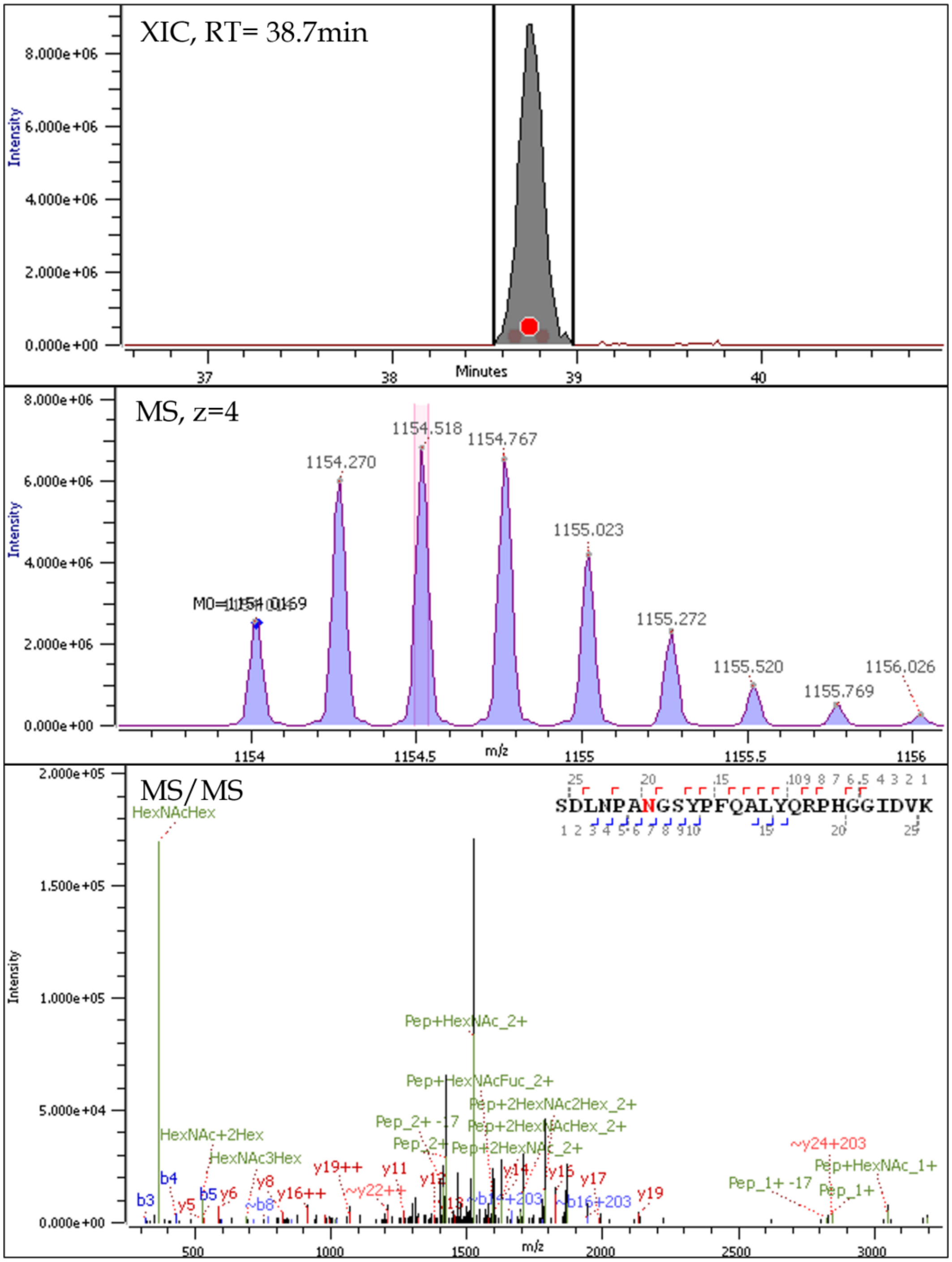

Figure S.18. Representative extracted ion chromatogram (XIC), mass spectrum (MS), and annotated tandem mass spectrum (MS/MS) for the identified peptide exhibiting glycosylation at residue N474.

T3 Glycopeptide: LP$T_{(HexNAc1Hex1)}$ QGPGR

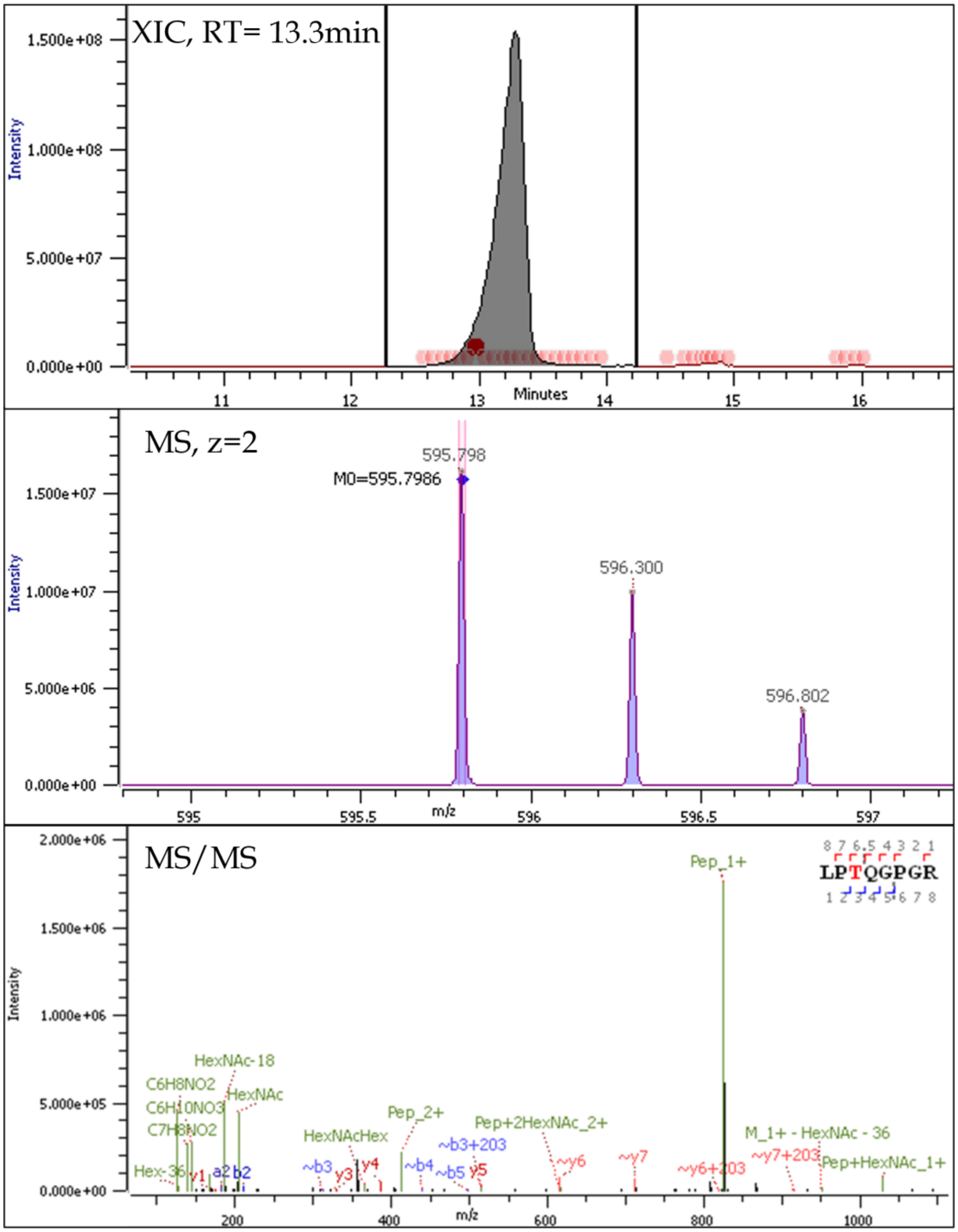

Figure S.19. Representative extracted ion chromatogram (XIC), mass spectrum (MS), and annotated tandem mass spectrum (MS/MS) for the identified peptide exhibiting glycosylation at residue T3.

T3 Aglycosylated Peptide: LPTQGPGR

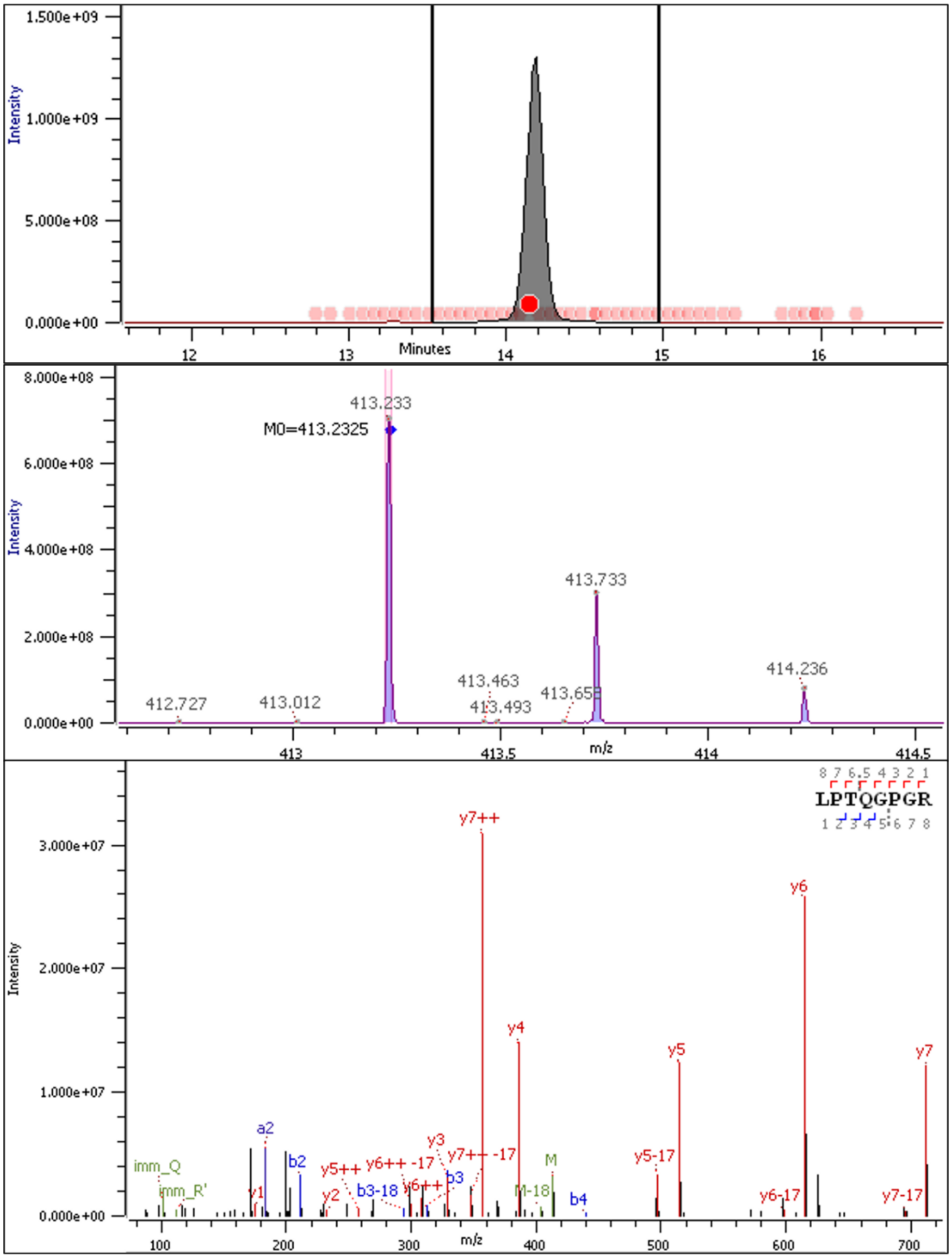

Figure S.20. Representative extracted ion chromatogram (XIC), mass spectrum (MS), and annotated tandem mass spectrum (MS/MS) for the identified peptide exhibiting no glycosylation at residue T3.

H39 Phosphorylated Peptide:
LVDGI**H**$_{\textbf{(phospho)}}$PYAVAWANLTN$_{\text{(HexNAc2Hex4, Man4)}}$AIR

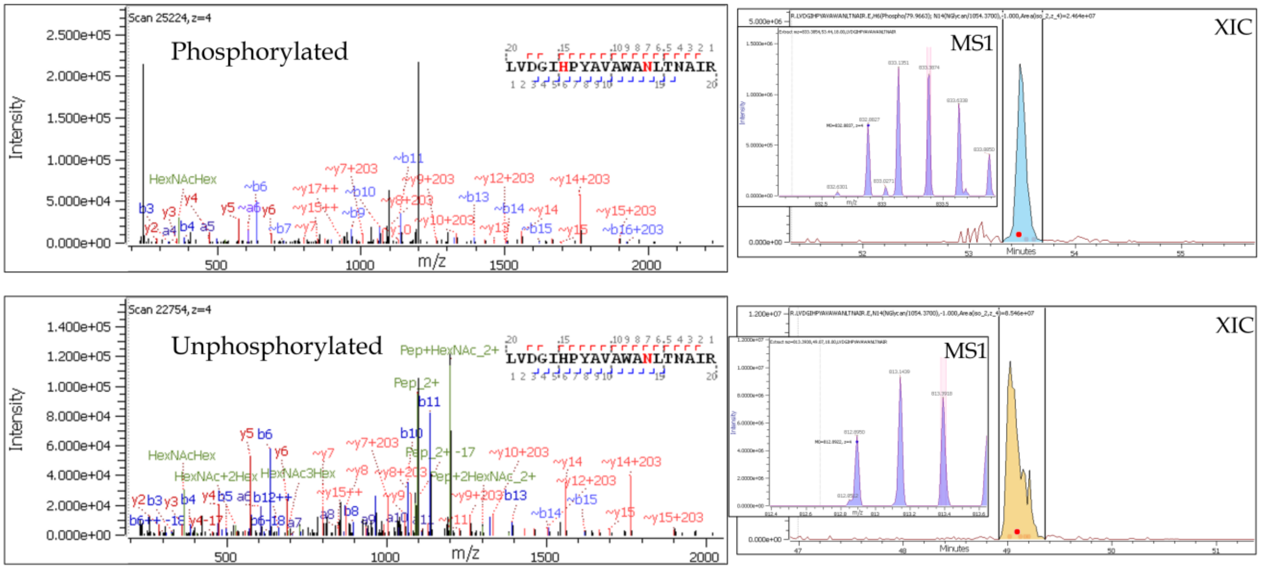

Figure S.21. Representative extracted ion chromatogram (XIC), mass spectrum (MS), and annotated tandem mass spectrum (MS/MS) for the identified peptide exhibiting phosphorylation and no phosphorylation at residue H39.

H487 Phosphorylated Peptide:
SDLN$_{\text{(HexNAc3Fuc1Hex6, Man6F+N)}}$PANGSYPFQALYQRP**H**$_{\textbf{(phospho)}}$GGIDVK

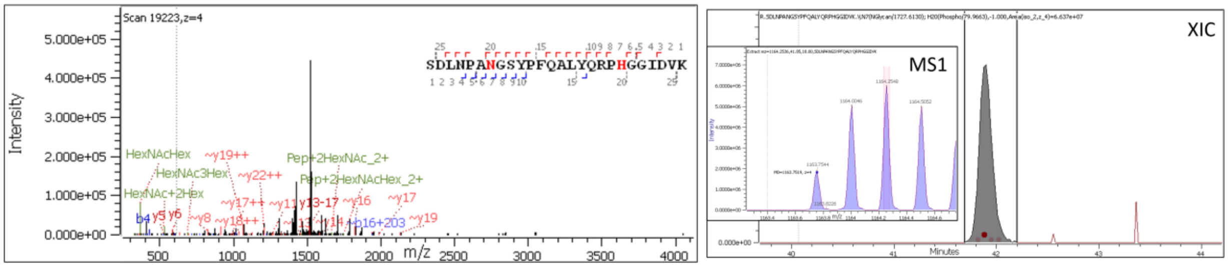

Figure S.22. Representative extracted ion chromatogram (XIC), mass spectrum (MS), and annotated tandem mass spectrum (MS/MS) for the identified peptide exhibiting phosphorylation at residue H487. Note that the unphosphorylated H487, Man6F+N glycopeptide was not observed. However, other glycoforms of the same phosphorylated peptide were observed (data not shown).

S85 Phosphorylated Peptide:
ETGWAYLDLGTNGSYN(HexNAc2Fuc2Hex3, Man3F2)DSLQAYAAGVVEASV**S(phospho)**
EELIYMHWMNTMVNYCGPFEYEVGYCEK

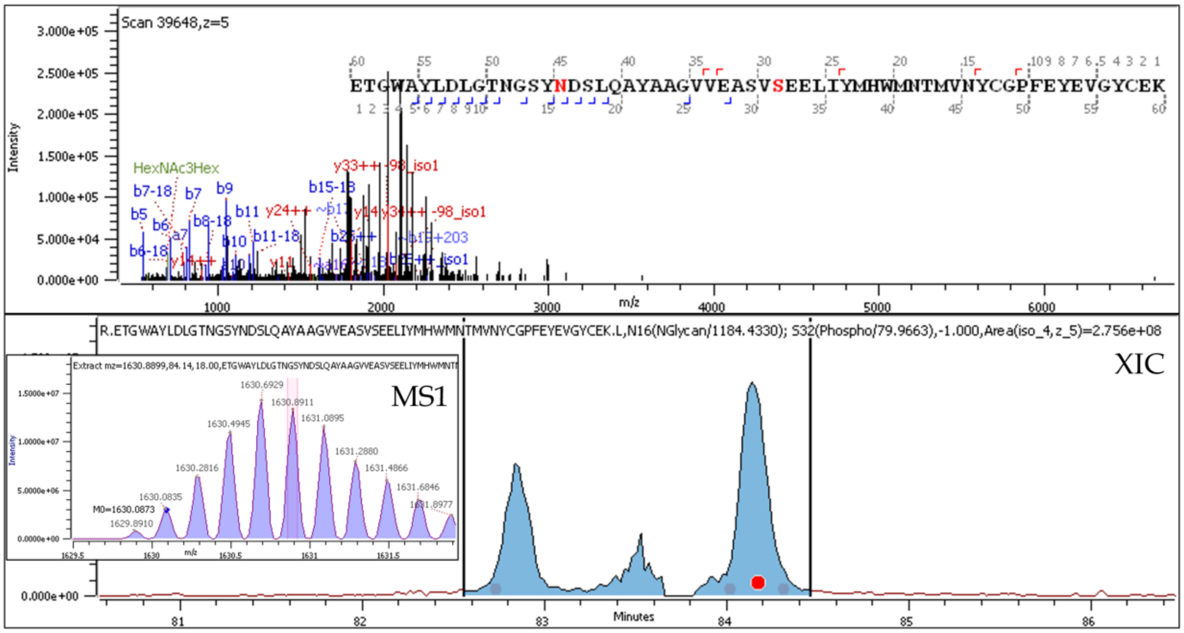

Figure S.23. Representative extracted ion chromatogram (XIC), mass spectrum (MS), and annotated tandem mass spectrum (MS/MS) for the identified peptide exhibiting phosphorylation at residue S85. Note that the unphosphorylated S85, Man3F2 glycopeptide was not observed. However, other glycoforms of the same phosphorylated peptide were observed (data not shown).

S468 Phosphorylated Peptide: $\mathrm{S_{(phospho)}DLNPAN_{(HexNAc2Hex6,\ Man6)}GSYPFQALYQR}$

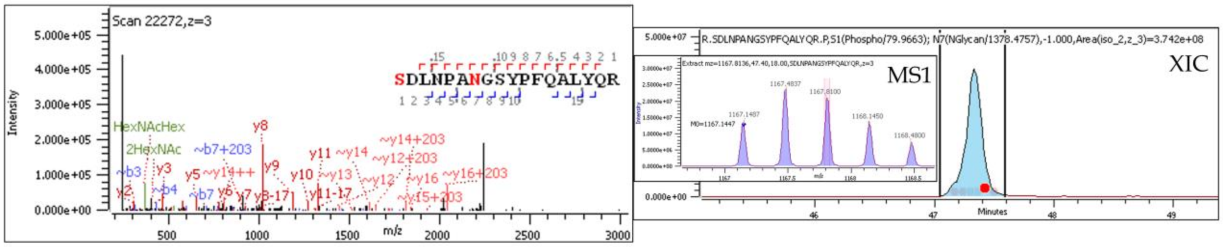

Figure S.24. Representative extracted ion chromatogram (XIC), mass spectrum (MS), and annotated tandem mass spectrum (MS/MS) for the identified peptide exhibiting phosphorylation at residue S85. Note that the unphosphorylated S468, Man6 glycopeptide was not observed. However, other glycoforms of the same phosphorylated peptide were observed (data not shown).

T167 Phosphorylated Peptide:

F**T**$_{\textbf{(phospho)}}$IKPLGFLLLQIAGDLEDLEQALN$_{\text{(HexNAc2Hex5, Man5)}}$K

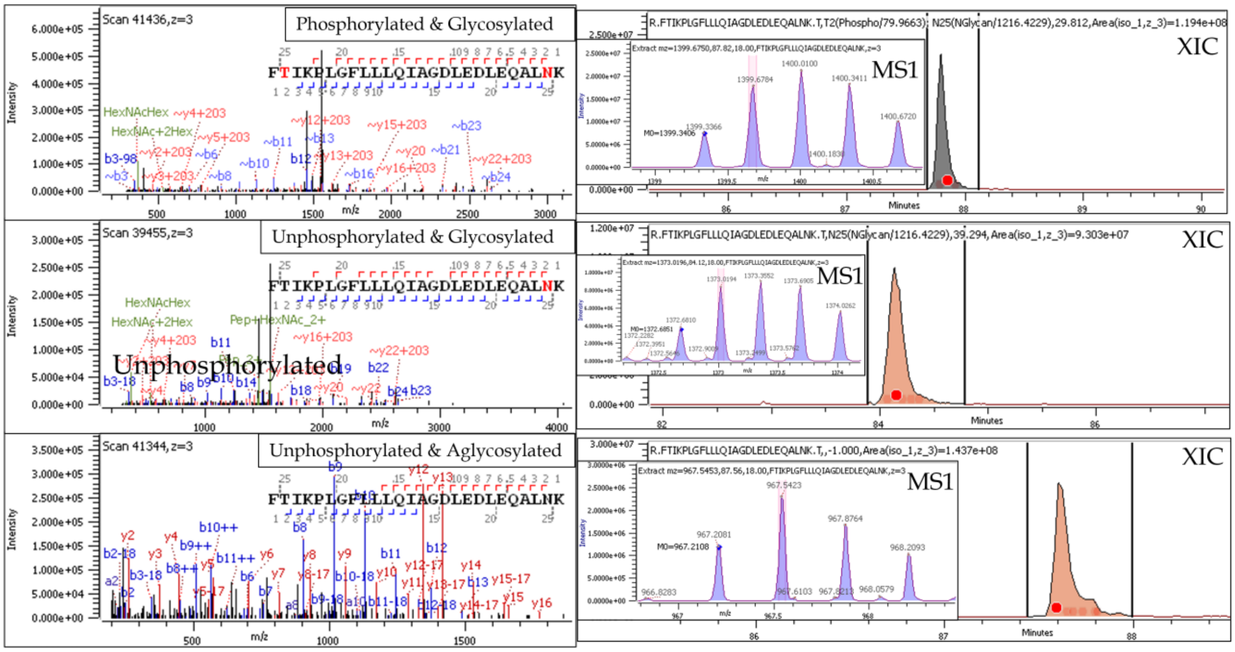

Figure S.25. Representative extracted ion chromatogram (XIC), mass spectrum (MS), and annotated tandem mass spectrum (MS/MS) for the identified peptide exhibiting phosphorylation and no phosphorylation at residue T167.

(a)

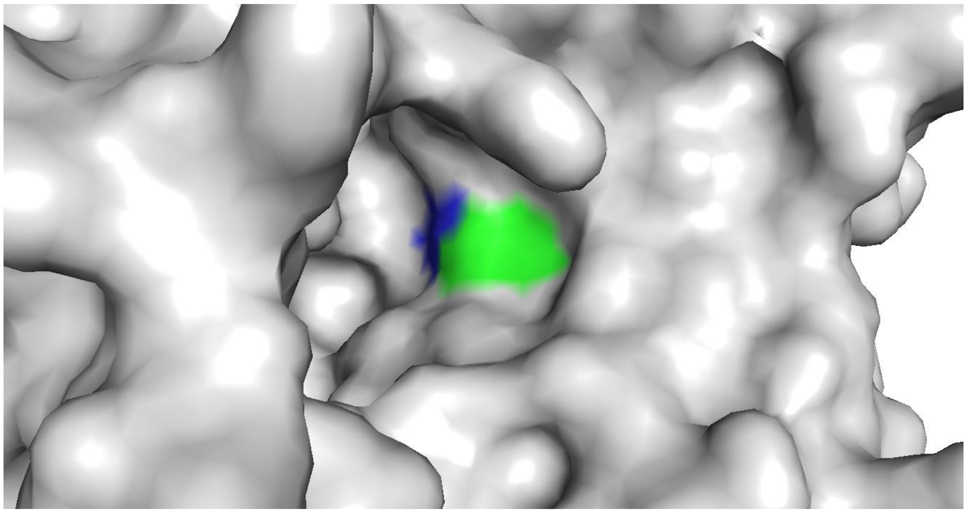

(b)

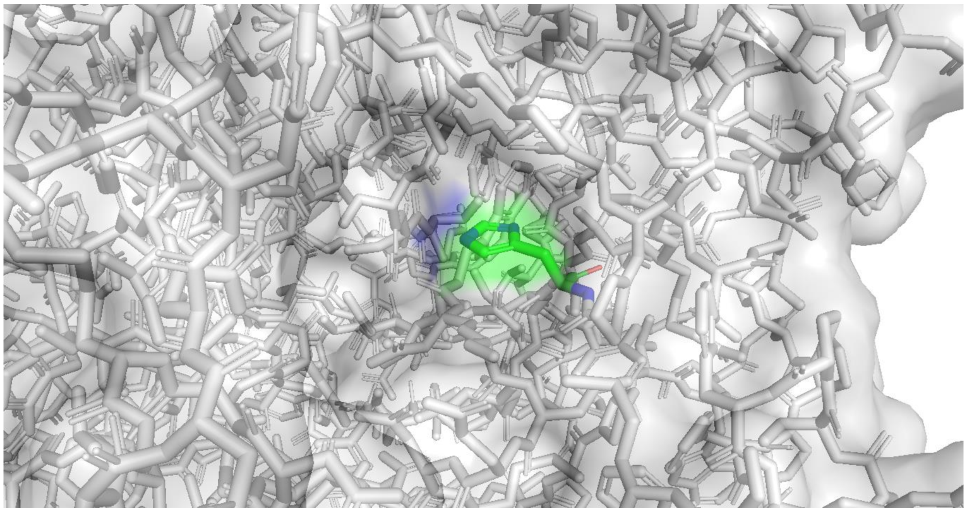

Figure S.26. Analysis of residue N47 of intact CHO PLBL2 using a homology model developed via AlphaFold2. N47 shown via (a) space-filling model and (b) semi-transparent surface, which demonstrates that N47 resides on the surface of CHO PLBL2 and is solvent exposed.

(a)

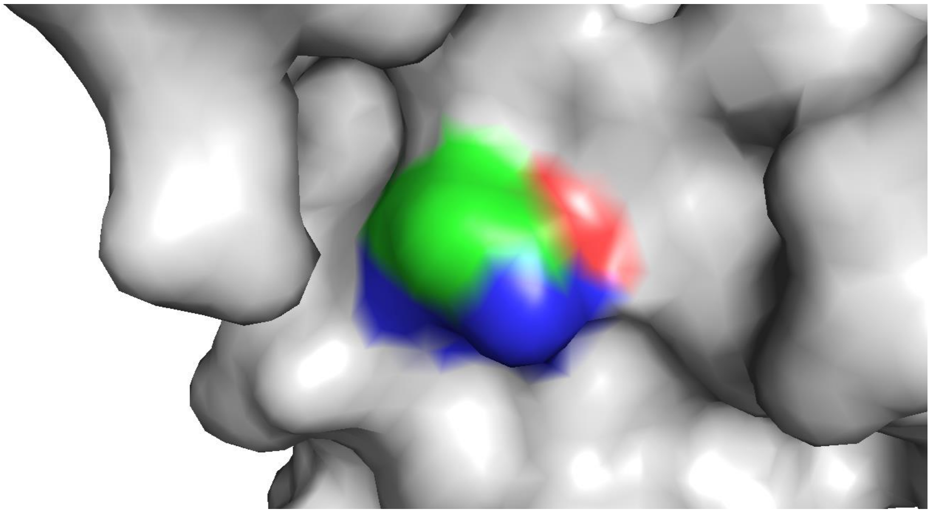

(b)

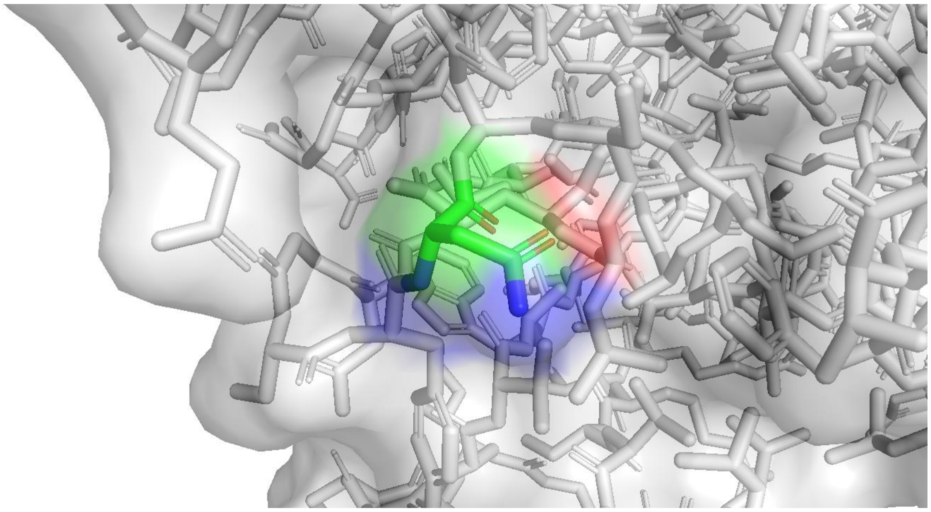

Figure S.27. Analysis of residue N69 of intact CHO PLBL2 using a homology model developed via AlphaFold2. N69 shown via (a) space-filling model and (b) semi-transparent surface, which demonstrates that N69 resides on the surface of CHO PLBL2 and is solvent exposed.

(a)

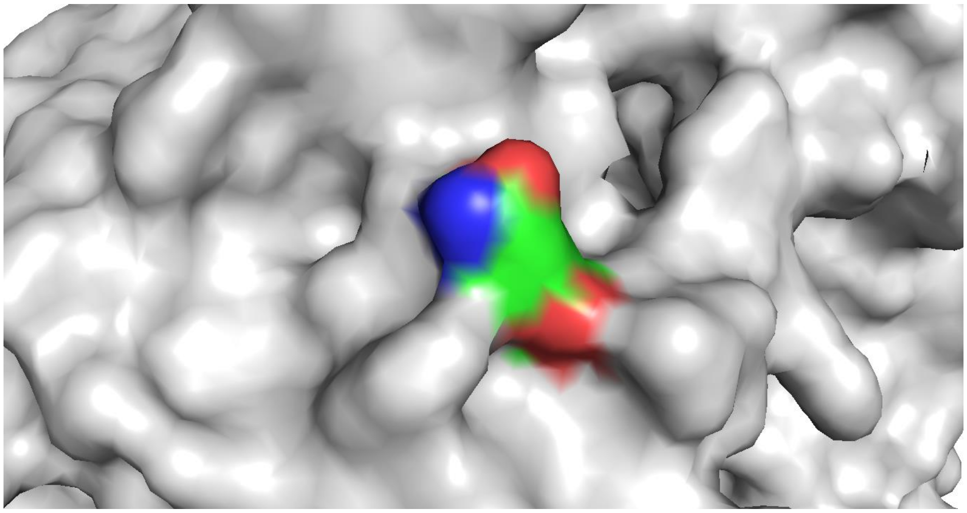

(b)

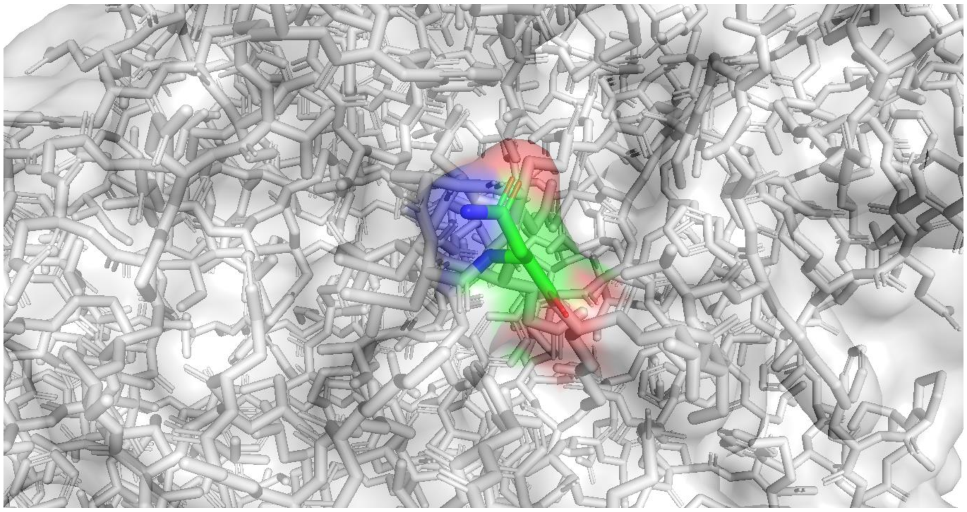

Figure S.28. Analysis of residue N190 of intact CHO PLBL2 using a homology model developed via AlphaFold2. N190 shown via (a) space-filling model and (b) semi-transparent surface, which demonstrates that N190 resides on the surface of CHO PLBL2 and is solvent exposed.

(a)

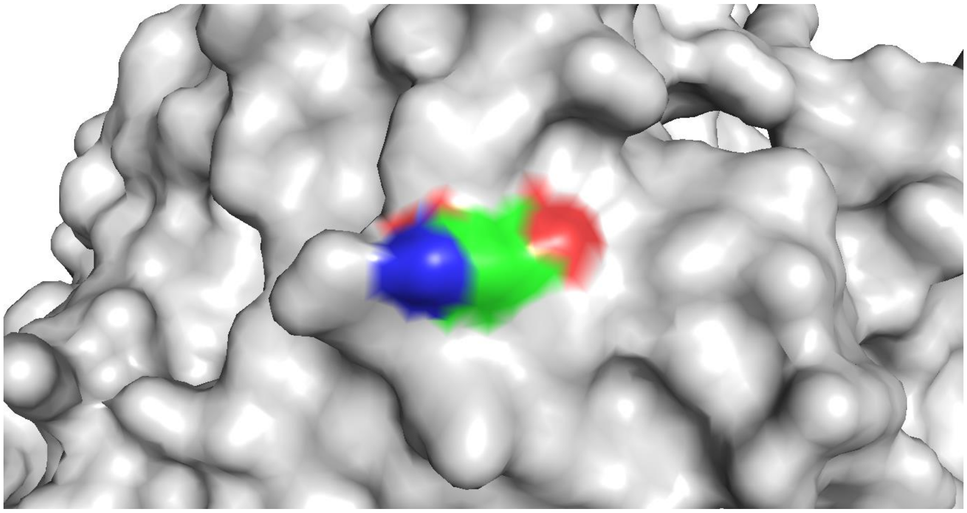

(b)

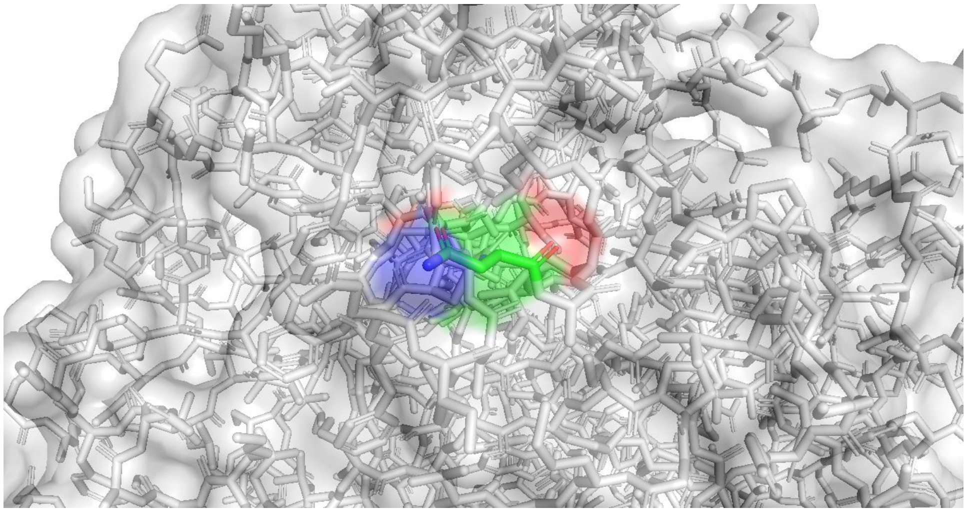

Figure S.29. Analysis of residue N395 of intact CHO PLBL2 using a homology model developed via AlphaFold2. N395 shown via (a) space-filling model and (b) semi-transparent surface, which demonstrates that N395 resides on the surface of CHO PLBL2 and is solvent exposed.

(a)

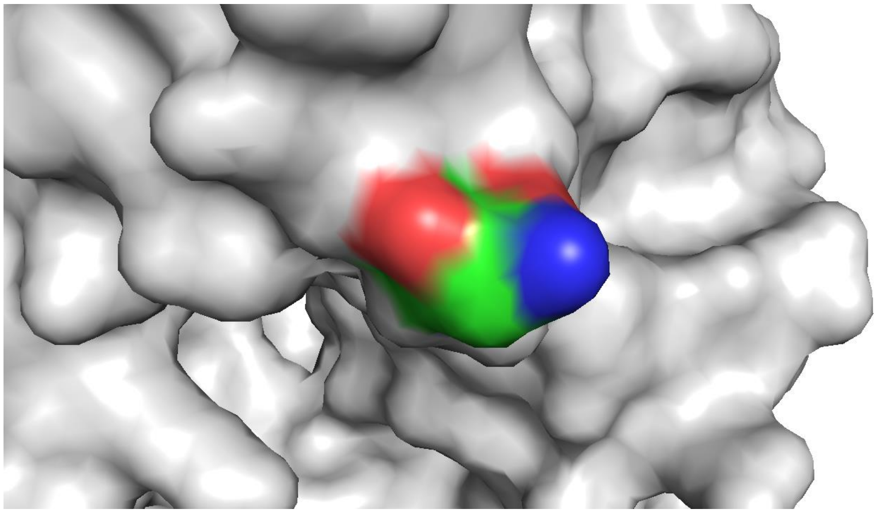

(b)

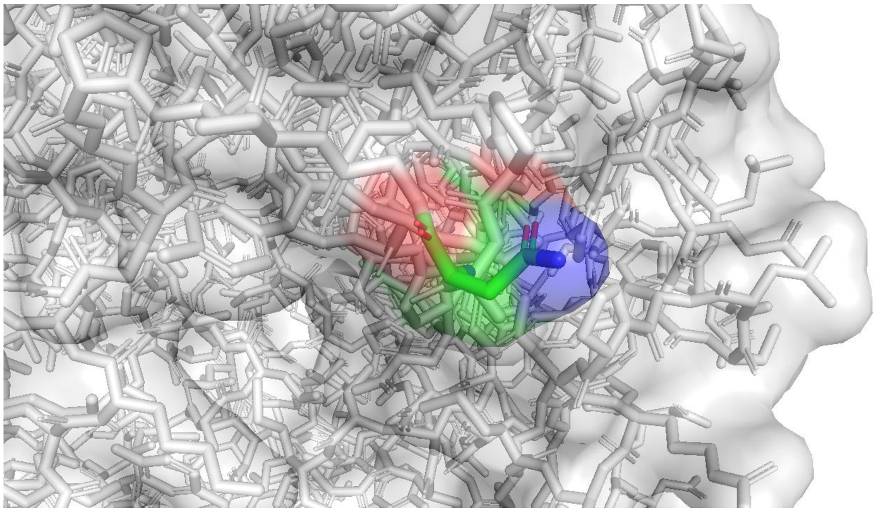

Figure S.30. Analysis of residue N474 of intact CHO PLBL2 using a homology model developed via AlphaFold2. N474 shown via (a) space-filling model and (b) semi-transparent surface, which demonstrates that N474 resides on the surface of CHO PLBL2 and is solvent exposed.

(a)

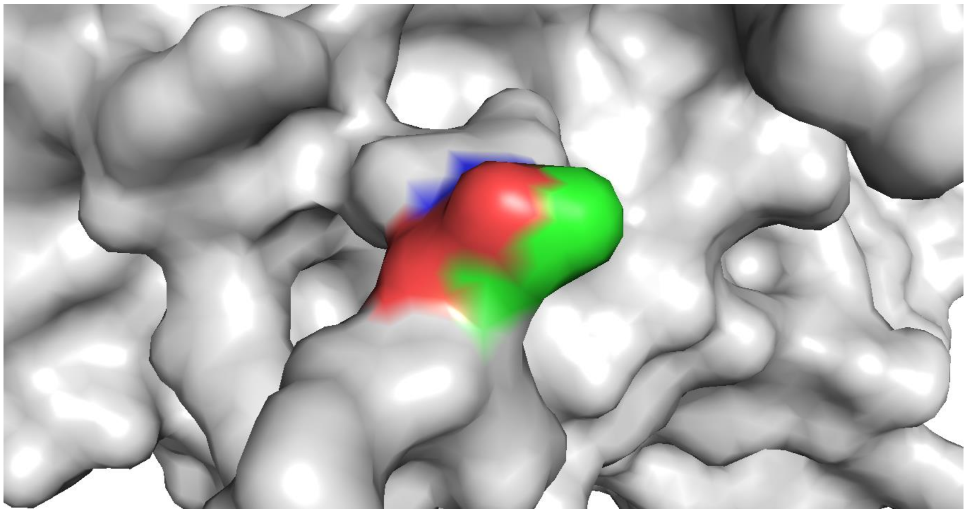

(b)

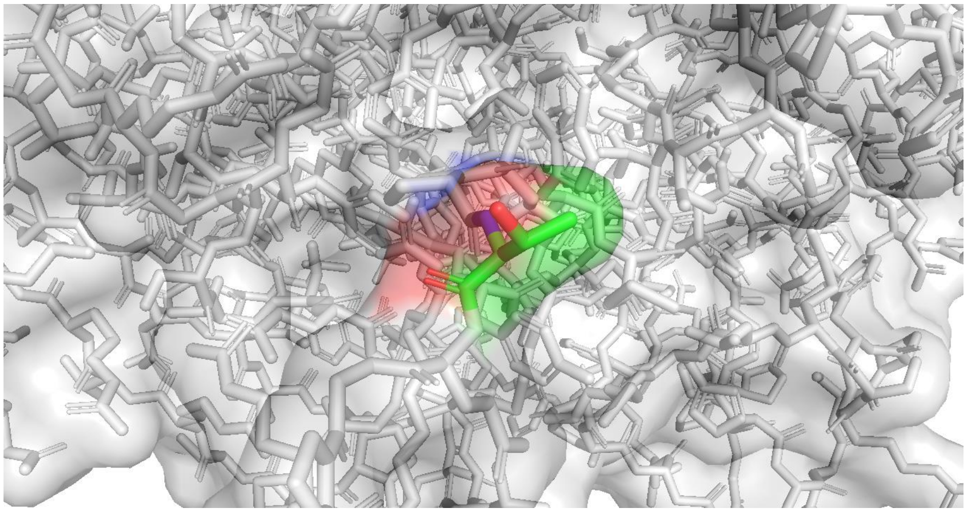

Figure S.31. Analysis of residue T3 of intact CHO PLBL2 using a homology model developed via AlphaFold2. T3 shown via (a) space-filling model and (b) semi-transparent surface, which demonstrates that T3 resides on the surface of CHO PLBL2 and is solvent exposed.

(a)

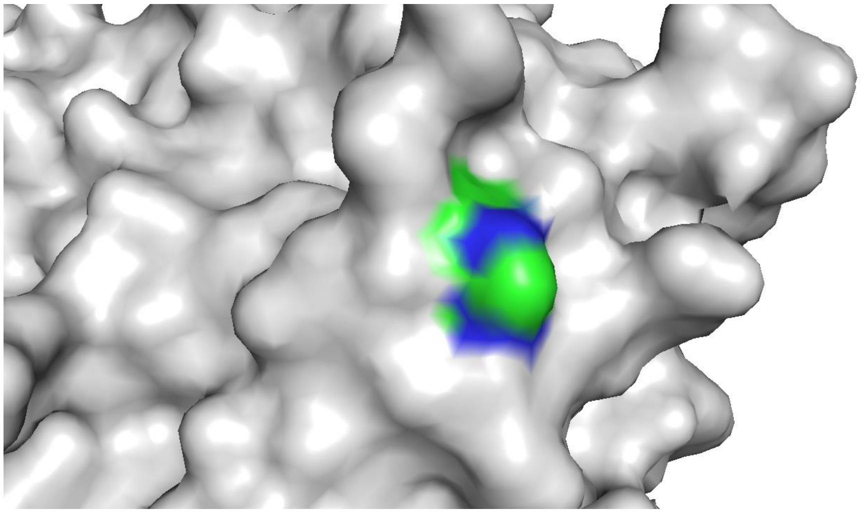

(b)

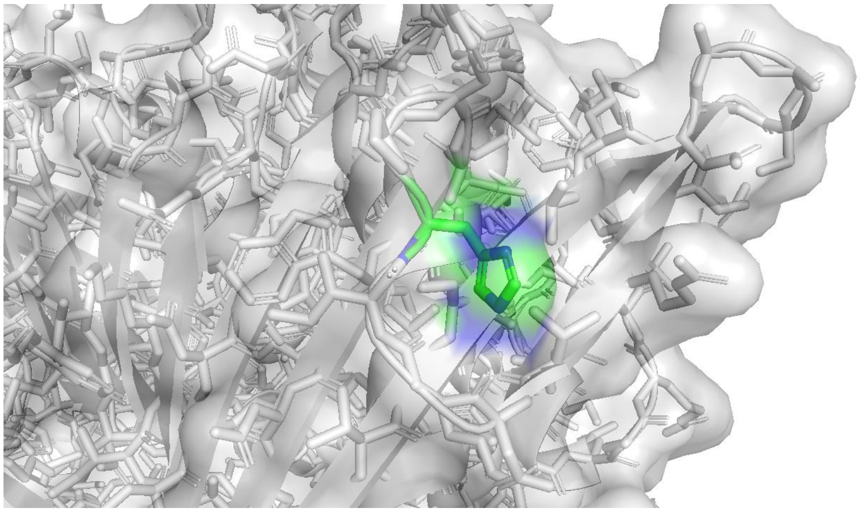

Figure S.32. Analysis of residue H39 of intact CHO PLBL2 using a homology model developed via AlphaFold2. H39 shown via (a) space-filling model and (b) semi-transparent surface, which demonstrates that H39 resides on the surface of CHO PLBL2 and is solvent exposed.

(a)

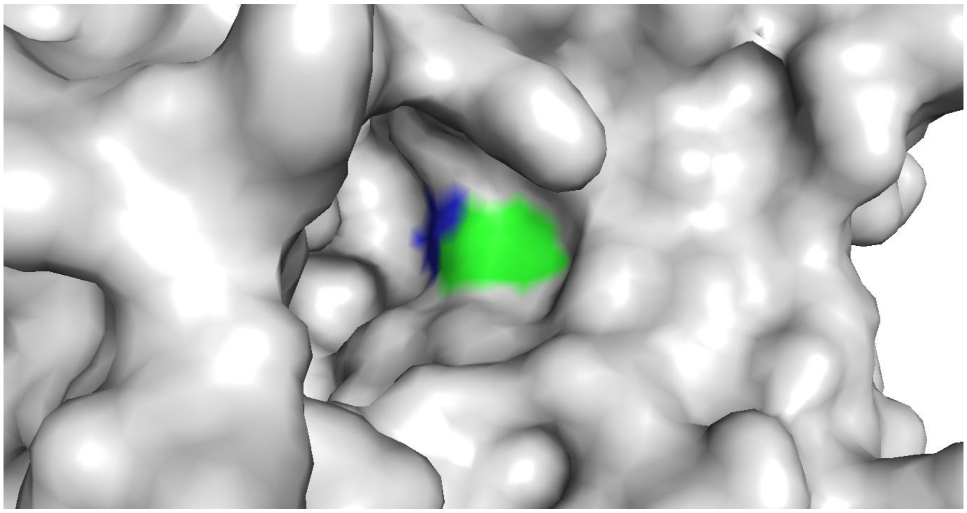

(b)

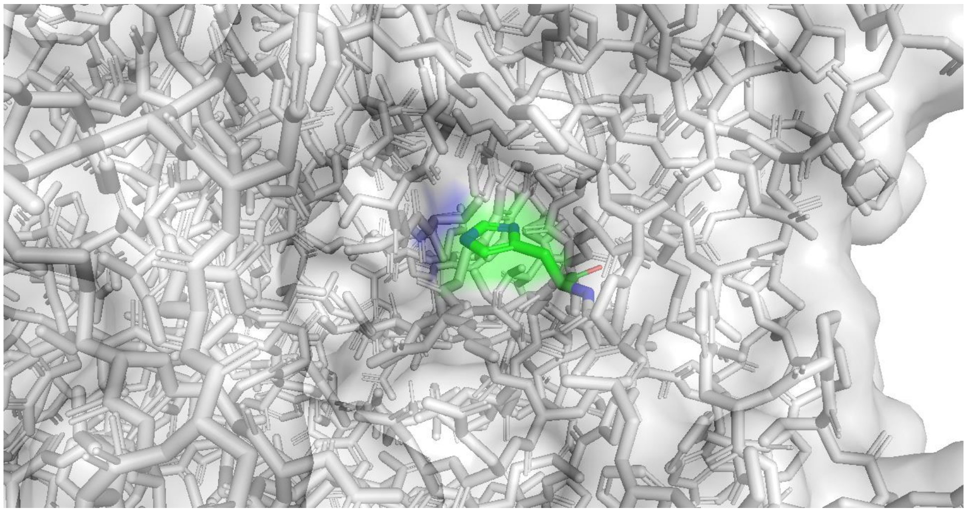

Figure S.33. Analysis of residue H487 of intact CHO PLBL2 using a homology model developed via AlphaFold2. H487 shown via (a) space-filling model and (b) semi-transparent surface, which demonstrates that H487 resides on the surface of CHO PLBL2 and is solvent exposed.

(a)

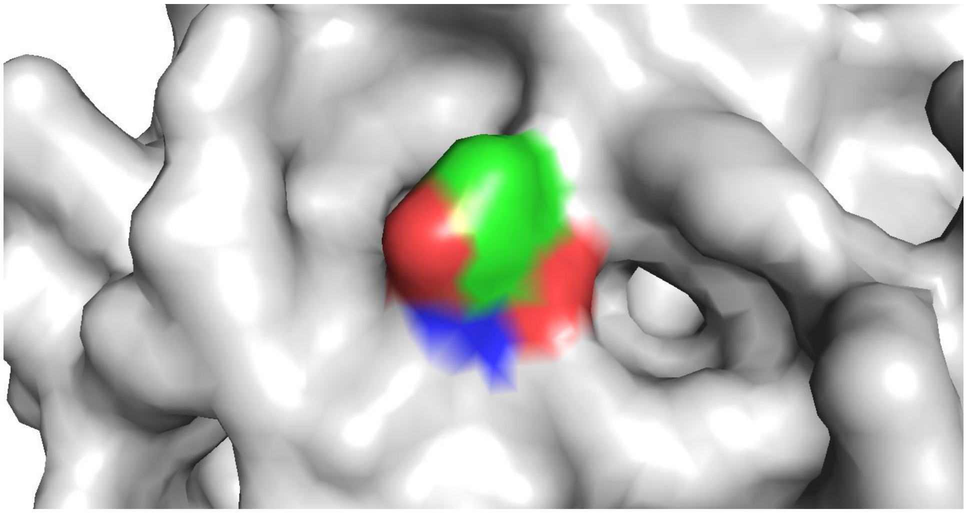

(b)

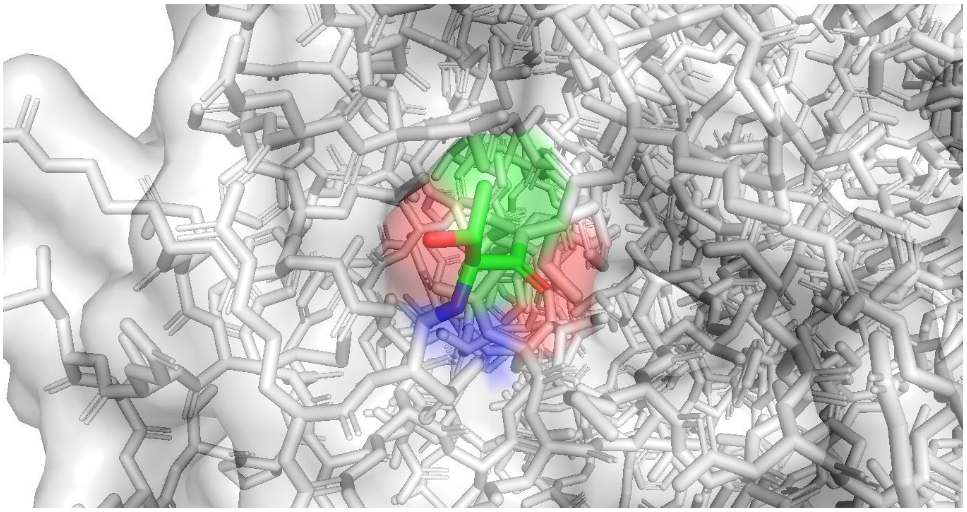

Figure S.34. Analysis of residue T167 of intact CHO PLBL2 using a homology model developed via AlphaFold2. T167 shown via (a) space-filling model and (b) semi-transparent surface, which demonstrates that T167 resides on the surface of CHO PLBL2 and is solvent exposed.

(a)

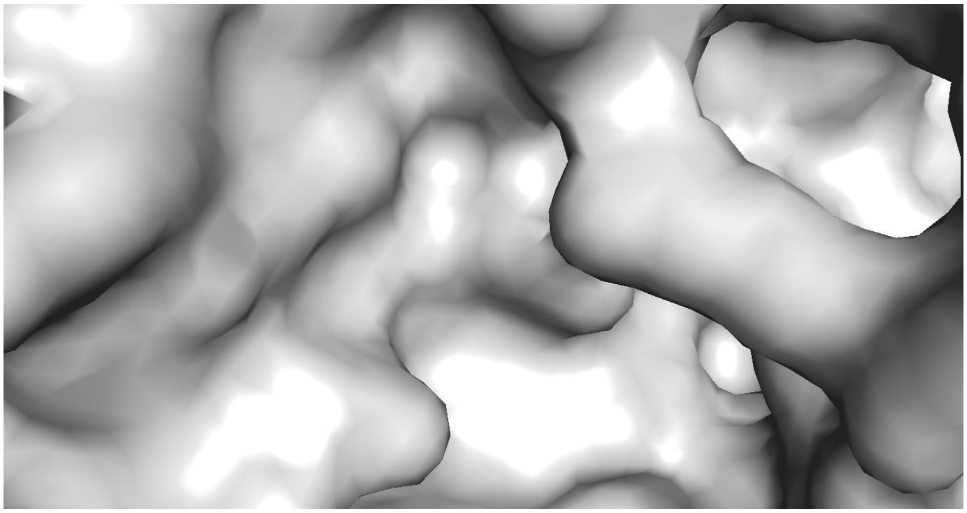

(b)

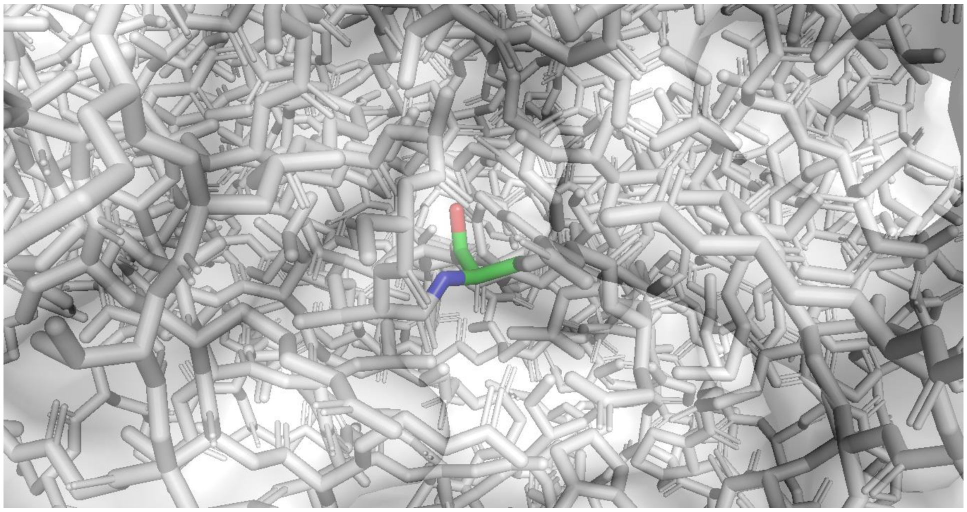

Figure S.35. Analysis of residue S85 of intact CHO PLBL2 using a homology model developed via AlphaFold2. S85 shown via (a) space-filling model and (b) semi-transparent surface, which demonstrates that S85 is buried within CHO PLBL2 and therefore not solvent exposed.

(a)

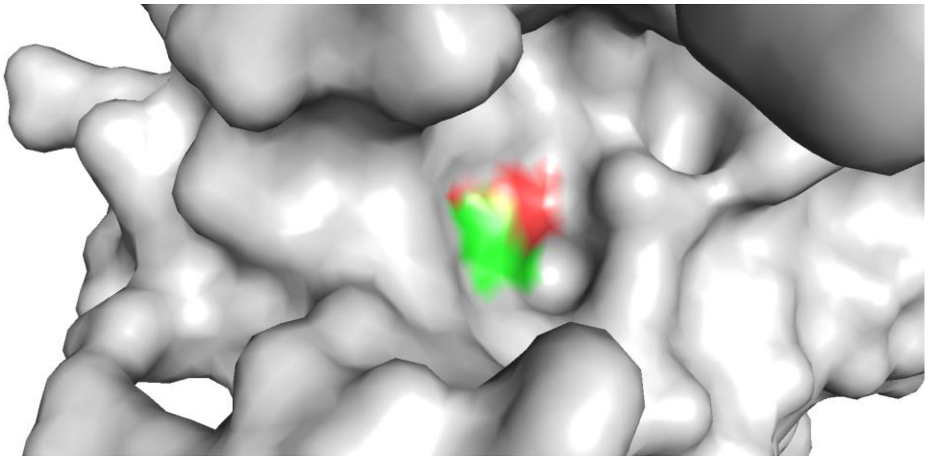

(b)

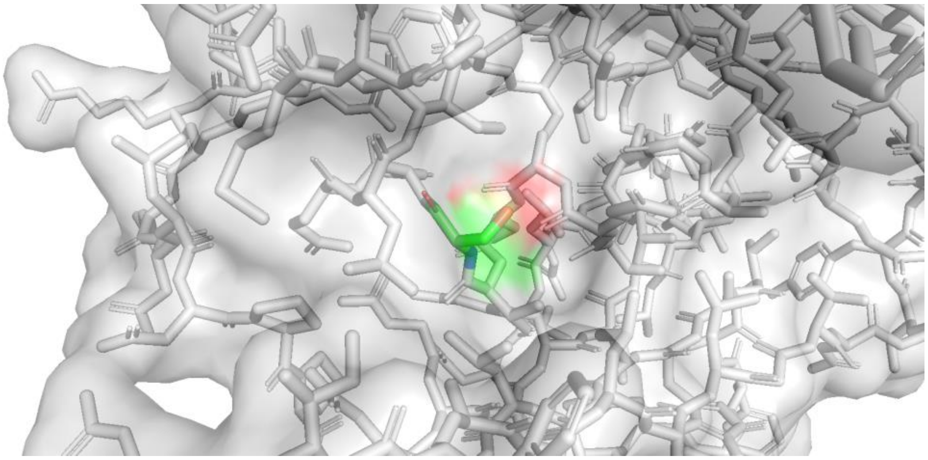

Figure S.36. Analysis of residue S85 of the N-terminal fragment of CHO PLBL2 using a homology model developed via AlphaFold2. S85 shown via (a) space-filling model and (b) semi-transparent surface, which demonstrates that that S85 resides on the surface of the N-terminal fragment after cleavage.

(a)

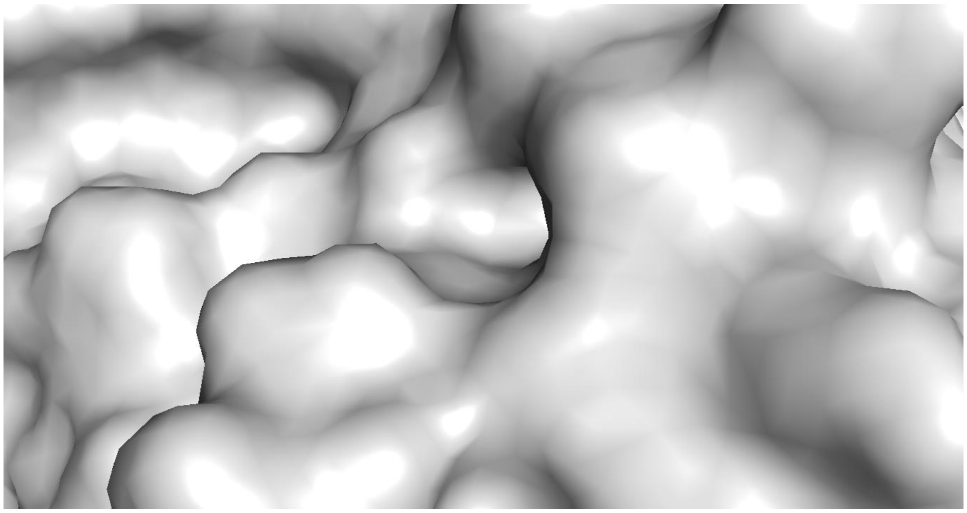

(b)

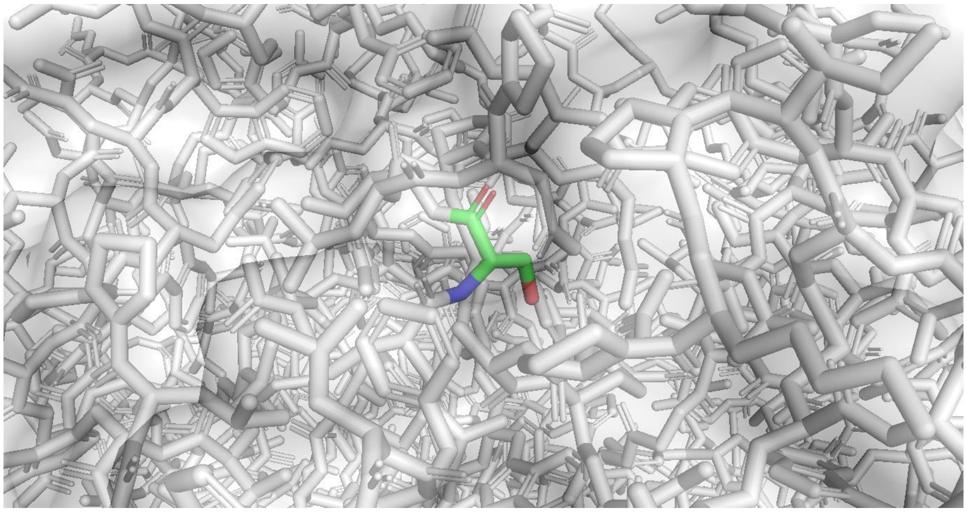

Figure S.37. Analysis of residue S468 of intact CHO PLBL2 using a homology model developed via AlphaFold2. S468 shown via (a) space-filling model and (b) semi-transparent surface, which demonstrates that S468 is buried within CHO PLBL2 and therefore not solvent exposed.

(a)

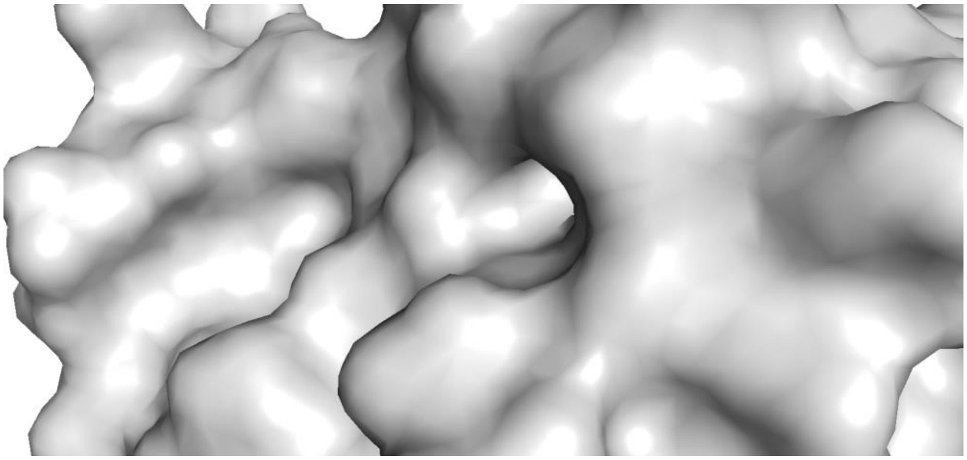

(b)

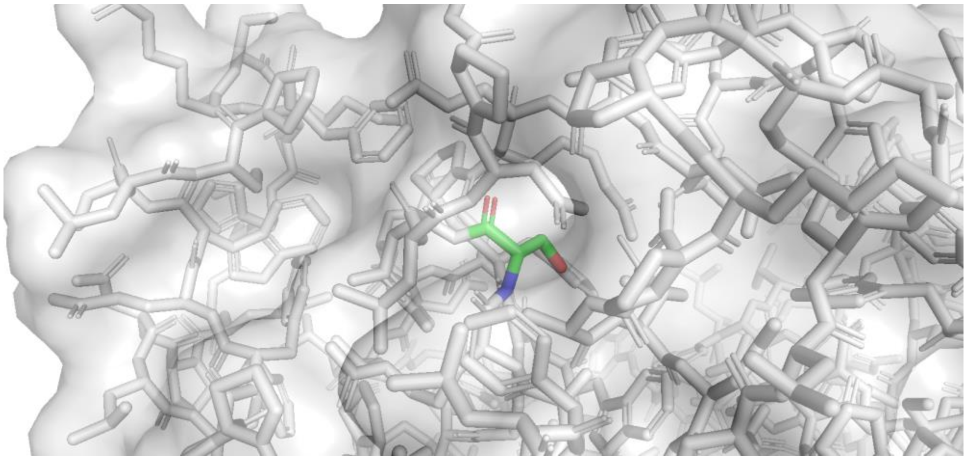

Figure S.38. Analysis of residue S468 of the C-terminal fragment of CHO PLBL2 using a homology model developed via AlphaFold2. S468 shown via (a) space-filling model and (b) semi-transparent surface, which demonstrates that S468 is buried within the C-terminal fragment after cleavage and therefore not solvent exposed.

References:

Dolan, M. E., Sadiki, A., Wang, L. L., Wang, Y., Barton, C., Oppenheim, S. F., & Zhou, Z. S. (2024). First site-specific conjugation method for native goat IgG antibodies via glycan remodeling at the conserved Fc region. *Antib Ther, 7*(3), 233-248. doi:10.1093/abt/tbae014
